# Parallel evolution of highly conserved plastid genome architecture in red seaweeds and seed plants

**DOI:** 10.1186/s12915-016-0299-5

**Published:** 2016-09-02

**Authors:** JunMo Lee, Chung Hyun Cho, Seung In Park, Ji Won Choi, Hyun Suk Song, John A. West, Debashish Bhattacharya, Hwan Su Yoon

**Affiliations:** 1 Department of Biological Sciences, Sungkyunkwan University, Suwon, 16419 Republic of Korea; 2 School of Biosciences 2, University of Melbourne, Parkville, VIC 3010 Australia; 3 Department of Ecology, Evolution and Natural Resources, Rutgers University, New Brunswick, NJ 08901 USA

**Keywords:** Plastid genome architecture, Parallel evolution, Rhodophyta, Florideophyceae, Seed plants

## Abstract

**Background:**

The red algae (Rhodophyta) diverged from the green algae and plants (Viridiplantae) over one billion years ago within the kingdom Archaeplastida. These photosynthetic lineages provide an ideal model to study plastid genome reduction in deep time. To this end, we assembled a large dataset of the plastid genomes that were available, including 48 from the red algae (17 complete and three partial genomes produced for this analysis) to elucidate the evolutionary history of these organelles.

**Results:**

We found extreme conservation of plastid genome architecture in the major lineages of the multicellular Florideophyceae red algae. Only three minor structural types were detected in this group, which are explained by recombination events of the duplicated rDNA operons. A similar high level of structural conservation (although with different gene content) was found in seed plants. Three major plastid genome architectures were identified in representatives of 46 orders of angiosperms and three orders of gymnosperms.

**Conclusions:**

Our results provide a comprehensive account of plastid gene loss and rearrangement events involving genome architecture within Archaeplastida and lead to one over-arching conclusion: from an ancestral pool of highly rearranged plastid genomes in red and green algae, the aquatic (Florideophyceae) and terrestrial (seed plants) multicellular lineages display high conservation in plastid genome architecture. This phenomenon correlates with, and could be explained by, the independent and widely divergent (separated by >400 million years) origins of complex sexual cycles and reproductive structures that led to the rapid diversification of these lineages.

**Electronic supplementary material:**

The online version of this article (doi:10.1186/s12915-016-0299-5) contains supplementary material, which is available to authorized users.

## Background

Eukaryotes acquired the photosynthetic organelle, the plastid, from a cyanobacterium through primary endosymbiosis that was followed by its integration as an intracellular organelle [1]. This double membrane-bound photosynthetic compartment [2] is widely believed to have had a single origin in the common ancestor of the Archaeplastida that comprises glaucophytes, red algae (Rhodophyta), and Viridiplantae (including green algae and land plants) [3, 4]. Despite the maintenance of many core components of the protein translation apparatus, photosystem, and ATPase complexes, the long-term impacts of Muller’s ratchet [5] has reduced the size of plastid genomes to ~100–200 kbp with some exceptional cases [e.g., the parasitic red alga *Choreocolax polysiphoniae* (90 kbp) and the non-photosynthetic land plants *Epipogium roseum* (19 kbp) and *E. aphyllum* (30 kbp)] [6, 7]. This is in contrast to a size of several megabases that likely defined the genome of the cyanobacterial endosymbiont. Plastid genome reduction is explained by outright gene loss or intracellular transfer to the host nuclear genome through endosymbiotic gene transfer (EGT) [8–11].

Red algae comprise ~7100 species found primarily in marine environments, although some also occur in freshwater habitats. Beyond their important ecological roles, red algae are crucial to the evolution of marine phytoplankton. This is because a single or, potentially, multiple ancient red algal lineages donated their plastid to a myriad of chlorophyll *c*-containing forms such as haptophytes, cryptophytes, stramenopiles, dinoflagellates, and apicomplexans through secondary (or additional rounds of) endosymbiosis [12–14]. Due to the importance of these chlorophyll *c*-containing groups as primary producers and grazers, a large number (currently 75) of species with red algal-derived plastids have been sequenced. However, the donor lineage of these plastids remains relatively poorly studied, with only 29 plastid genomes reported, and these primarily from a single red algal class, the sexually reproducing (with one exception, see below) Florideophyceae, with no genomes available from three other classes that rely primarily on asexual reproduction (Stylonematophyceae, Compsopogonophyceae, and Rhodellophyceae) [6, 15–27]. This imbalance in available data is readily apparent when compared to Viridiplantae, for which hundreds of complete plastid genomes have been determined. These “green” plastid genomes have been used to resolve basal group relationships in Viridiplantae and to document the high genome architecture variability in most green algae when compared to the extreme conservation found in flowering plants (about 800 genomes in GenBank) [28–30].

Given their shared evolutionary history, red algae and Viridiplantae provide an ideal test bed to compare plastid genome architecture and the extent of gene loss and EGT over >1 billion years of evolution. To enable this analysis, we determined 17 novel red algal plastid genomes with three additional datasets derived from partial plastid genomes, bringing the total to 48 for this phylum. These genomes represent most red algal classes as well as the 12 orders of the largest subclass Rhodymeniophycidae (5011 species) in the class Florideophyceae (6755 species; see Algaebase: http://www.algaebase.org). With these broadly sampled genome data we asked the following two questions: what are the major trends in gene loss and EGT in these taxa, and what can we learn about the evolution of genome architecture following the ancient split of the red and green lineages within the Archaeplastida? Our results demonstrate extensive variation in algal plastid gene content and genome architecture but identify highly conserved plastid genomes in Florideophyceae and seed plants. We speculate that the independent origins of complex sexual cycles may have constrained the evolution of these latter genomes.

## Results and discussion

### General features of red algal plastid genomes

Seventeen complete red algal plastid genomes were determined using next-generation sequencing (NGS) methods (Additional file 1: Table S1). An additional three partial genome datasets were added for the phylogenetic analysis. Genome size and gene contents varied at the class and species level. For instance, plastid genome size in the early-diverging red algal class Cyanidiophyceae was smaller (145–167 kbp) than in the other classes: Stylonematophyceae (*Bangiopsis subsimplex*, 204 kbp), Compsopogonophyceae (*Erythrotrichia carnea*, 210 kbp; *Rhodochaete parvula*, 222 kbp), Porphyridiophyceae (*Porphyridium purpureum*, 217 kbp; *P. sordidum*, 259 kbp), and the well-supported monophyletic group comprising Bangiophyceae (187–196 kbp) and Florideophyceae (167–194 kbp). An average of 202 protein-coding sequences (cds) was found in these plastid genomes with some variation in cds number and the encoded gene inventory (e.g., there were 184 cds in *Galdieria sulphuraria* and *Hildenbrandia rivularis*, whereas there were 235 cds in *Grateloupia taiwanensis* and 224 cds in *P. purpureum*) (Additional file 1: Tables S1 and S2). Most of the early-diverged classes contained two copies of the ribosomal DNA operon (rDNA), whereas single or partially inactivated (pseudogenes) duplicated rDNAs were found in most florideophycean species (Additional file 1: Table S1). This suggests the independent loss of one copy of the rDNA operon in these taxa. Duplicated rDNA operons (or inverted repeat including rDNAs) are broadly distributed in all primary and secondary plastids as well as in cyanobacterial genomes [31–34]. Most red algal plastid genomes contained ~30 transfer RNAs (tRNAs), except *G. sulphuraria* (39 tRNAs) and nine Bangiophyceae species (35–37 tRNAs). The Stylonematophyceae, Porphyridiophyceae, and Compsopogonophyceae had intron-rich (38–65 introns) plastid genomes that distinguished them from other red algae (i.e., two introns in the *trn*Me tRNA and *chl*B genes from the Florideophyceae). Most of these introns were predicted to be members of the Group II intron family (Additional file 1: Table S3) based on RNAweasel (http://megasun.bch.umontreal.ca/cgi-bin/RNAweasel/RNAweaselInterface.pl) analyses. Interestingly, a homologous group of intronic open-reading frames (ORFs) was related to the intronic ORFs of *trn*Me tRNA in all florideophycean species. Phylogenetic analysis of these sequences (Additional file 2: Figure S1) indicated that the intronic ORFs were derived from prokaryotes (including cyanobacteria) and spread into different genes (i.e., *dna*K, *psa*A, *atp*B, *psb*E, *rpo*C2, *inf*C, and *glt*B) in early-diverging red algae. Nine bangiophycean species likely underwent independent losses of this region.

### Phylogeny and genome architecture of red algal plastid genomes

Several aspects of the red algal tree of life remain unresolved. These include the relationships among: 1) the basal classes, 2) the 12 orders of Rhodymeniophycidae, and 3) the nine orders of Nemaliophycidae [35–37]. These relationships have now been clarified using the maximum likelihood (ML) tree (Additional file 3: Figure S2) that was inferred using a dataset of 191 concatenated plastid protein-coding genes (Additional file 1: Table S4) with the outgroup taxa in the class Cyanidiophyceae [38, 39]. Although there remain unresolved relationships among the early-diverged classes, the relationships of all 12 orders of Rhodymeniophycidae are well resolved (72–100 % ML bootstrap support, MLB), and monophyly of the three nemaliophycidaen orders are well supported (100 % MLB).

Based on this multigene phylogeny, we studied structural variation among red algal plastid genomes using MUMmer [40] with the Gracilariales species *Gracilaria tenuistipitata* as the reference. This analysis showed that plastid genome architecture in the classes Cyanidiophyceae, Stylonematophyceae, Compsopogonophyceae, and Porphyridiophyceae are highly diverged when compared to the conserved architecture in the classes Bangiophyceae and Florideophyceae (Additional file 3: Figure S3). Cyanidiophyceae show high within-class structural variation (Additional file 3: Figure S4) when compared to Bangiophyceae and Florideophyceae (Additional file 3: Figure S3). The plastid genomes of the basal lineages (Stylonematophyceae, Compsopogonophyceae, and Porphyridiophyceae) represent a wide swath of red algal diversity and have retained a large number of introns (Additional file 1: Table S1). To test the effect of the intron data on our results, we excluded these intervening regions from the analysis (Additional file 3: Figure S5b) and found that structural variation remained comparable between these two analyses (Additional file 3: Figure S5a).

Although plastid genomes in the class Bangiophyceae are conserved, it is important to note that they derive from a single order (Bangiales). In comparison, the diverse orders of the Florideophyceae, except the subclass Hildenbrandiophycidae (*H. rivularis*, *H. rubra*, and *Apophlaea sinclairii*), showed surprisingly highly conserved plastid genome architectures (Additional file 3: Figure S3). This latter result suggests that plastid genome synteny was constrained in the non-Hildenbrandiophycideae members of Florideophyceae.

### Independent convergent structural changes in major red algal plastid genomes

**Fig. 1:**
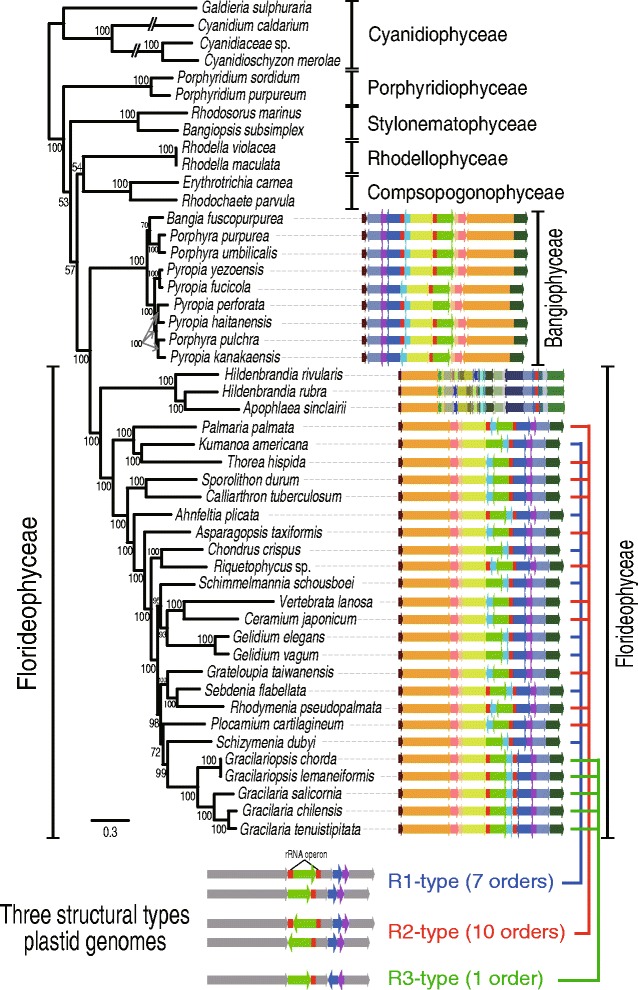
Red algal plastid genomes in the class Florideophyceae. In this synteny analysis of R1-type (seven orders), R2-type (10 orders), and R3-type (one order) structures, conserved color-schemes indicate a homologous plastid region except in the subclass Hildenbrandiophycidae, where synteny is not maintained, although the same color scheme applies. All of the *red* rectangular boxes indicate rDNA operons. Each color indicates a conserved genomic fragment. Under the tree, a simplified synteny image of the three different plastid genome types is shown in which the *gray arrows* indicate conservation and *green*, *blue*, and *purple* arrows indicate variable regions

The complete set of red algal plastid genomes was used to generate a highly resolved multigene tree for this phylum. Using this tree as reference, we found that most florideophycean species, except Hildenbrandiophycidae, have three different plastid genome architectures (Fig. 1) that we named R1- (Rhodophyta-type 1), R2-, and R3-type. R1- and R2-types co-occurred in multiple places in the tree, even within highly supported monophyletic clades. For example, within the well-supported Nemaliophycidae (100 % MLB), two R2-types (*Palmaria* and *Thorea*) and one R1-type (*Kumanoa*) were present. The same result was found for the *Chondrus* (R1-type) and *Riquetophycus* (R2-type) monophyletic clade (100 % MLB). The R3-type was limited to the five Gracilariales species. To determine if these plastid types define convergence or, alternatively, poorly resolved nodes in the phylogenetic tree, every species with the R1- and R2-type architecture was artificially enforced into a monophyletic group. Thereafter, we used the approximately unbiased (AU) test [41, 42] to determine if the enforced tree was significantly better, or worse, or not different from the original topology. Our results show that the enforced tree topology was significantly worse and was rejected at *p*-value = 1*e* ^−20^. This suggests that the structural types evolved in a convergent manner in most florideophycean species.

### Origins of the R1-, R2-, and R3-type plastid genome architectures

**Fig. 2:**
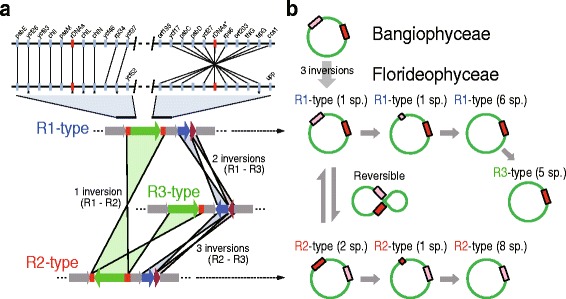
Synteny analysis and proposed evolutionary pathway for generating the three types of plastid genomes. **a** Syntenic differences between the three types of plastid genome structure in the Florideophyceae and comparison with the R1-type plastid genome in Bangiophyceae. *Asterisks* (*) indicate variable sites due to gene loss or pseudogenization. The *chl*L and *chl*N genes are present only in the plastid genomes of the subclass Nemaliophycidae. **b** Proposed evolutionary pathway for generating the three plastid genome types in Florideophyceae. The *green circles* indicate the plastid genome and the *red and pink rectangular boxes* show the rDNA operon(s). The number of species containing duplicated rDNAs, semi-duplicated rDNAs, and single rDNA operons are shown in parentheses

Differences between the R1- and R2-type plastid genome architectures encompassed one inversion between the rDNA operons, whereas two inversions were found between R1- and R3-types, and three inversions were found between the R2- and R3-types (Fig. 2a). Because the bangiophycean lineage and R1-type species have a similar gene synteny in the flanking region of the two rDNA operons, and because one inversion is present between the R1- and R2-types internal to the two rDNA operons, we postulate that the R1-type is ancestral and evolved into the R2-type and R3-type. Because complementary rDNA operon sequences can recombine during circular chromosomal division, it is likely that the origins of the R1- and R2-type orientations were mediated by the rDNA operons (Fig. 2b). It was interesting to note that two rDNA operons were retained in *Ahnfeltia* (R1-type), and *Palmaria* and *Rhodymenia* (R2-type), but were pseudogenized (i.e., partial sequences) in *Sebdenia* (R1-type) and *Riquetophycus* (R2-type), or one copy completely lost in six R1-type and eight R2-type species (Figs. 1 and 2b and Additional file 1: Table S1). Because Muller’s ratchet [5, 43] acts on plastid genomes (i.e., leading to unidirectional loss), it is likely that each structural type was fixed along with the loss of one rDNA operon in red algal plastid genomes (Fig. 2b). This process could lead to structural stabilization of plastid DNA and is likely to be convergent, because the same plastid genome architecture was found in phylogenetically distantly related species.

### Parallel events in plant plastid genome evolution

We found that plastid genome reduction and structural conservation characterized seed plants (angiosperms and gymnosperms). Based on a preliminary survey of structural variation among the available 703 flowering plant (angiosperm) plastid genomes (see Additional file 1: Tables S5 and S6 and Additional file 4: Figures S6–S15), we chose 62 (46 orders and one un-ranked taxa) that included all orders in this angiosperm clade (sampling details are shown in “Methods”). In addition, 20 plastid genomes were sampled from other Viridiplantae lineages, including five gymnosperms (from 67 available), three pteridophytes (from 18 available), two bryophytes (from 12 available), five charophytes (from 11 available), and five green algae (from 58 available).

**Fig. 3:**
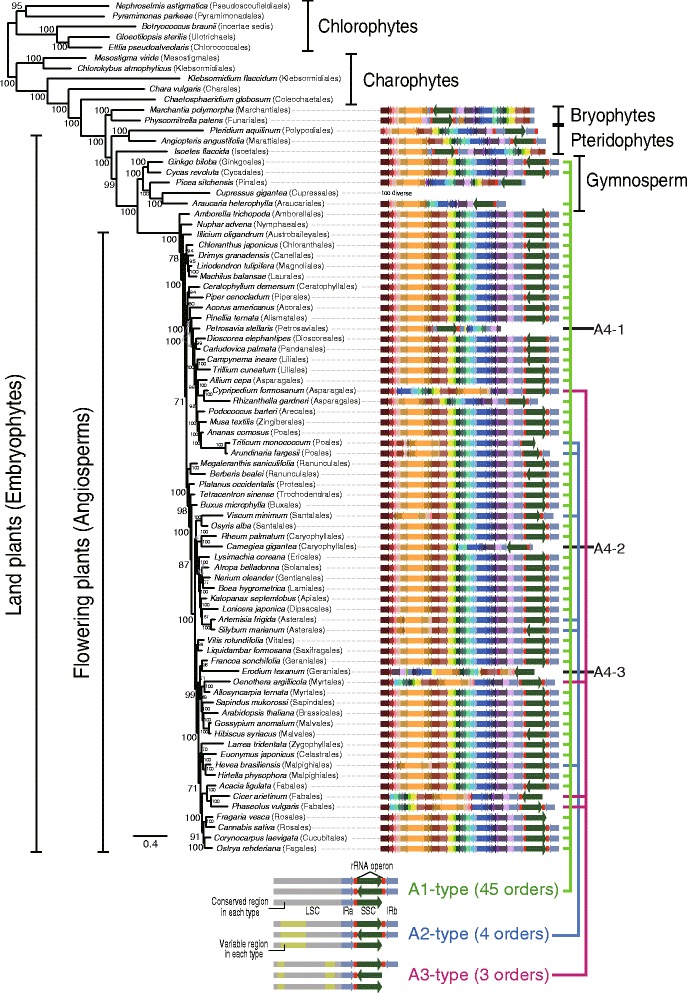
Synteny of the three major (A1–A3) and one independent (Rest) type of plastid genome architecture in the Viridiplantae. In this diagram of the A1-type (45 orders), A2-type (four orders), A3-type (three orders), and the Rest-type (three orders) in the seed plants, the shared colors indicate homologous plastid regions. The *red rectangular boxes* indicate ribosomal DNA operons and the *red boxes and their flanking light blue arrows* indicate inverted repeat (IR) regions. Between the IR regions, the *dark green arrow* indicates the small single copy region in plastid genomes. Each color indicates a conserved genomic fragment. Under the tree, a simplified synteny image of the three types plastid genomes are shown, whereby the *gray regions* indicate conservation and *light green* indicates variable regions

Structural variation in angiosperm plastid genomes was inferred using *Ostrya rehderiana* as the reference, due to its conserved, canonical architecture. The minor rearrangements we found in these genomes are shown in Fig. 3, which presents a ML tree made using a concatenated dataset of 77 plastid genes (Fig. 3, Additional file 1: Table S7, and Additional file 4: Figure S6). Green algal and charophyte plastid genomes were highly variable in architecture (Additional file 4: Figures S7 and S8). In contrast, bryophyte, pteridophyte, gymnosperm, and angiosperm genomes showed high conservation (Additional file 4: Figures S9–S14). Within the flowering plants, three major genome types (A1–A3; named for angiosperm types) were identified, as well as several atypical cases (about 40 structural types were collectively denoted as the Rest-type), based on a comparison of the large single copy (LSC) and small single copy (SSC) regions and the direction and presence/absence of inverted repeats (IRs). The A1-type (49 species from 45 orders; Additional file 4: Figure S11) was most widespread in the phylogeny, with the A2-type (six species from four orders; Additional file 4: Figure S12), A3-type (four species from three orders; Additional file 4: Figure S13), and Rest-type (three species from three orders; Additional file 4: Figure S14) being sporadically distributed among taxa, as described above for Florideophyceae plastid genome types. Inversions of the entire SSC were present in all four types, together with the absence of the IR, but this was highly variable in the Rest-type (i.e., *Petrosavia stellaris*, *Carnegiea gigantea*, *Erodium texanum*, see Fig. 3). IR loss occurred in nine species of seed plants but only three cases also had inversion of the SSC (A1: *Fragaria vesca*; A2: *Triticum monococcum*; and A3: *Cicer arietinum*). This heterogeneity of IR-containing plastid genomes and subsequent loss of the IR through homologous recombination (heteroplasmy, when present in individuals and populations) [44–53] appears to have occurred in both the red and green lineages (see Figs. 1 and 3). The frequency of IR degeneration is, however, different between plastid genomes of most florideophycean species and flowering plants: ~80 % (19 out of 24) red algal plastid genomes lost one rDNA operon, whereas 2.5 % (18 out of 703) angiosperm species lost (or inactivated) the rDNA operon located in one IR region (437 A1-type, 181 A2-type, 28 A3-type, and 57 Rest-type) (Additional file 1: Table S5 and Additional file 4: Figures S11–S14).

Similar patterns of conserved genome architecture were found in other land plants, including gymnosperms, pteridophytes, and bryophytes (Additional file 4: Figures S9 and S10). For example, among the 67 gymnosperm plastid genomes, 18 different structural types were found including A1-type (14 species from the Cycadales, Araucariales, and Ginkgoales; Additional file 1: Table S6 and Additional file 4: Figure S10), which was the dominant form among angiosperms. About 4.4 % of gymnosperms (3 out of 67) have lost one IR region or it was pseudogenized. Among bryophytes (12 spp.) and pteridophytes (18 spp.), plastid genomes retain two IRs, and the genome architecture is highly conserved within each group (Additional file 4: Figure S9). In contrast, structural variation among chlorophytes (58 spp.) and charophytes (11 spp.) was too extensive to identify a single pattern (Additional file 4: Figures S7 and S8). A similar high level of variation was found among three sexually reproducing colonial volvocine species [*Gonium pectoral* (Goniaceae), and *Pleodorina starrii* and *Volvox carteri* (Volvocaceae)] (Additional file 4: Figure S15).

It is noteworthy that vastly different frequencies of IR loss were found among the two sister red and green lineages (i.e., 80 % in Florideophyceae versus 2.5 % in seed plants). These taxa have vastly different divergence times: that is, 781 million years ago (mya) for the major Florideophyceae and 318 mya for seed plants [54–57]. They also show different evolutionary trajectories: that is, the red lineage underwent degeneration of the IR region of cyanobacterial origin, resulting in the retention of only one rDNA operon with two tRNAs (i.e., *trn*A and *trn*I), whereas the green lineage underwent gradual expansion of the IR region (e.g., 38,398 bp in *Plantago media*) from the ancestral IR gene set found in charophytes, bryophytes, and pteridophytes (*trn*N, *tnr*R, *rrn*5, *rrn*4.5, *rrn*23, *trn*A, *trn*I, *rrn*16, and *trn*V) to the angiosperms with the additional gene set (*rps*12-3, *rps*7, *ndh*B, *trn*L, *ycf*2, *trn*H, *rpl*23, and *rpl*2) (see details in [53]). Interestingly, seed plants contain whirly protein and organelle-to-nucleus crosstalk genes, and suppress short IR-derived illegitimate recombination in plastid genomes [58]. Red algae lack these features [59].

Among the highly variable R-types (i.e., 40 atypical cases from 57 species, see Additional file 4: Figure S14), the plastid genome architectures of the Geraniaceae (Geraniales) have been extensively studied [60–62]. Within the Geraniales, there are about 11 different types without any single one being a typical structure (i.e., A1–A3), although the sister taxa of the Geraniales show the A1-type (e.g., *Francoa sonchifolia* and *Melianthus villosus*) (Additional file 5: Figure S16). To better understand this exceptionally high structural variation, we compared each genome with the ancestral A1-type and noted the breakpoint distance (BD). For instance, structural changes to accommodate the A1- to A2-type require four BDs, whereas the A1- to A3-type change needs three BDs (Additional file 5: Figure S16). Within the Geraniaceae, five *Pelargonium* species have two to five BDs with respect to the A1-type, whereas 10 *Erodium* species have three to eight BDs. Interestingly, *E. texanum* shows higher variation (13 BDs) than other *Erodium* species (e.g., six BDs in the strongly supported sister species *E. crassifolium*). Moreover, six *Erodium* species (*E. foetidum*, *E. rupestre*, *E. carvifolium*, *E. manescavi*, *E. reichardii*, and *E. trifolium*) show degeneration of one of the rDNA operons, suggesting structural fixation after the loss of repeat sequences. In contrast, four independent cases of extensive structural rearrangements (higher than 10 BDs) were found within the Geraniaceae (green blocks in Additional file 5: Figure S16). This high level of architectural variation could be impacted by the repeat sequences (i.e., recombination sites) as suggested in previous studies with co-occurring high nucleotide substitution rates [60–62]. Although it is not clear why and how this exceptionally high level of structural variation originated in the Geraniales, repeat sequences (i.e., duplicated rDNA operons and IR regions) within the plastid genome could be one of the factors that contributed to this phenomenon, as observed in the red and green lineages.

### Plastid genome variation in red algal-derived secondary plastids

To test plastid genome conservation in other photosynthetic groups, we studied all available plastid genome data from red algal-derived plastid groups, including seven brown algae (Phaeophyceae, stramenopiles), 20 diatoms (Bacillariophyceae, stramenopiles), seven Eustigmatophyceae (including six *Nannochloropsis* species, stramenopiles), four haptophytes, and four cryptophytes (Additional file 6: Figures S17–S21). Most of these plastid groups are poorly represented in public databases (e.g., seven Eustigmatophyceae, four haptophytes and cryptophytes); hence, it is difficult to reach a robust conclusion about their genome evolution. Nonetheless, we found relatively high genome conservation in brown and cryptophyte algae (Additional file 6: Figures S17 and S18). The brown algae show a rapid radiation and are a sexually reproducing, multicellular group [63] (2045 species; http://www.algaebase.org) with generally highly conserved plastid genome architectures among three orders (Additional file 6: Figure S17) [64, 65]. In contrast, diatoms show a relatively high amount of rearrangement among 13 orders (Additional file 6: Figure S19). Diatoms are a unicellular, sexual/asexual reproducing group, and underwent an explosive radiation that resulted in more than 200 genera and 100,000 extant species. In summary, analysis of red algal-derived plastid-containing groups shows that sexually reproducing, tissue-forming, multicellular lineages have stable plastid genome architectures.

### Comparison of Rhodophyta and Viridiplantae plastids

**Fig. 4:**
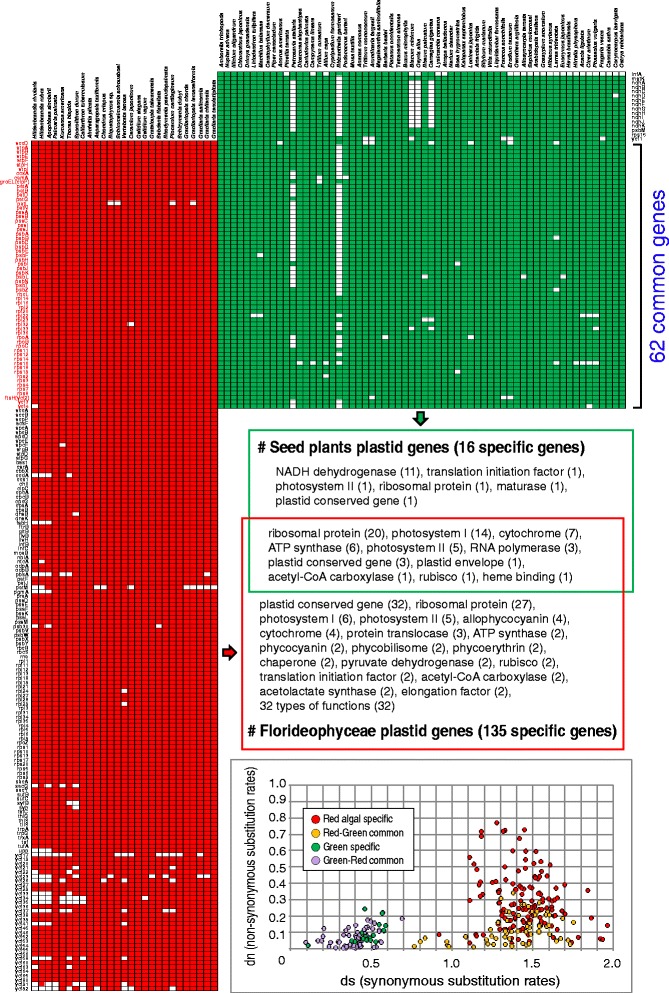
Comparison of major plastid gene sets between Florideophyceae (red algae) and seed plants (green plants). Functions and substitution rates are shown based on taxon sampling of the maximum likelihood trees (Figs. 1 and 3). Sixty two common genes are clustered based on their homology (*rpo*C1 and *rpo*C2 genes were combined as *rpo*C). Functional categories are shown based on UniProt and NCBI databases, and “plastid conserved gene” indicates *ycf* gene. Red algal-specific genes (135), green lineage-specific genes (16), and their common genes (62; red and green lineage) were analyzed by synonymous substitution rates (*ds*) and non-synonymous substitution rates (*dn*) and plotted as a graph

In both cases of red and green plastid genomes, basal unicellular and filamentous algal groups showed high structural variation, whereas a highly conserved architecture originated in parallel in the multicellular Florideophyceae (i.e., three structural types) and in seed plants (i.e., three structural types). This observation led us to test whether any common features are shared by florideophyte and seed plant plastid genomes. In seed plants, genome size varied from 19 to 217 kbp (median = 153 kbp) with 18–273 proteins (median = 84 proteins) and 28–51 % GC-content (median = 37 %), with some exceptional cases including highly reduced plastid genomes of non-photosynthetic plants (19 kbp in *Epipogium roseum* and 30 kbp in *E. aphyllum*), the biggest plastid genome size with a greatly expanded IR region (217 kbp in *Pelargonium x hortorum*), and the largest gene number (273 genes including unclassified ORFs in *Pinus koraiensis*) (Additional file 7: Figure S22) [7, 66]. In contrast, Florideophyceae generally have larger genomes (91–194 kbp in size; median = 181 kbp) encoding 71–235 proteins (median = 202), and lower GC-contents (median = 29 %). These lineages share only 62 homologous genes, including ribosomal proteins (20), photosystem-I (14), photosystem-II (5), cytochrome (7), and ATP synthase (6) (Fig. 4). However, 135 genes are present only in red algae, including conserved genes (32), ribosomal proteins (27), and several genes for pigments of allophycocyanin (4), phycocyanin (2), phycobilisomes (2), and phycoerythrin (2). Comparison of the rates of synonymous and non-synonymous substitution using the method of Nei and Gojobori [67] failed to show a correlation in values between shared and non-shared genes, although seed plants had lower rates for both classes of change (Fig. 4). This result suggests that plastid genomes in the red and green lineages underwent differential gene loss after their split from a putative single common ancestor [68]. The putative absence of organelle-to-nucleus crosstalk genes and whirly proteins [59] could in part explain the high gene content of red algal plastids (see also below). The shared and unique retained plastid genome sequences faced comparable selective constraints. Differential plastid gene loss has been reported in another more recent example of primary plastid endosymbiosis in *Paulinella* species after acquisition of the organelle from a *Synechococcus*-*Cyanobium* type of α-cyanobacterium [69].

### Orthologous gene families in Archaeplastida

**Fig. 5:**
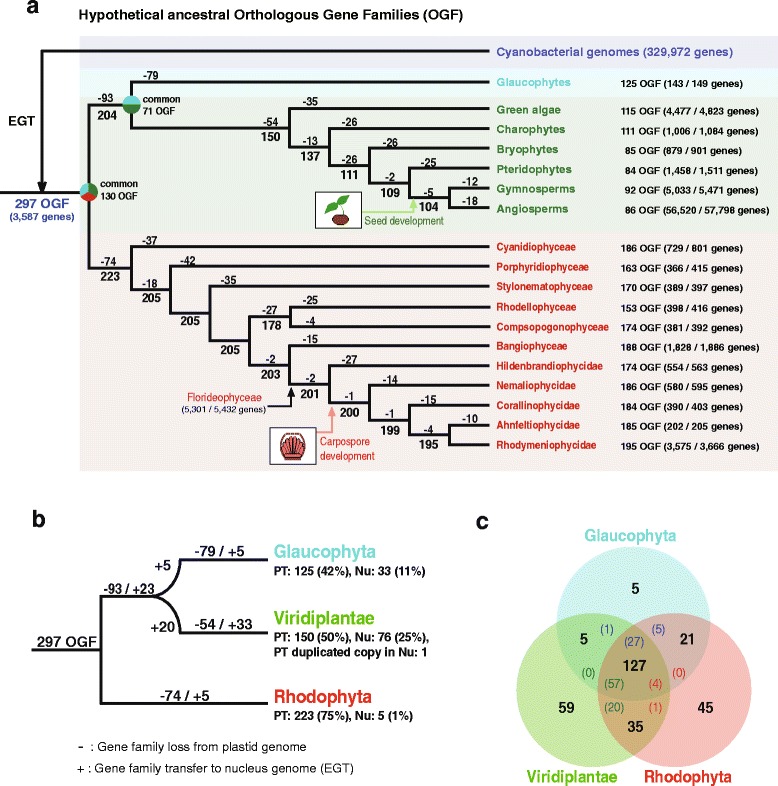
Analysis of orthologous gene families (OGFs) in the Archaeplastida. **a** Parsimony-based model of gene family evolution of primary plastid genes based on the reference phylogeny of these plastid genomes (Figs. 1 and 3 and Additional file 8: Figure S23). The 297 OGFs from 3587 cyanobacterial genes were predicted from a database of 329,972 cyanobacterial genes (99 cyanobacteria strains), and are regarded as hypothetical ancestral OGFs of primary plastid genes. The numbers of ancestral OGF homolog groups and genes in glaucophytes (*cyan*), green lineage (*green*), and red algae (*red*) plastid genomes are shown on the right of the taxon name. Each loss (-) and hypothetical ancestral stage of the OGF groups is shown in the tree based on parsimony. Common OGF groups between “ancestral stage of glaucophytes and green lineage” and “red algae” and between “glaucophytes” and “green lineage” are described in each ancestral clade. **b** Simplified map of gene family loss (-) from plastid genomes and gene transfer (+) to the nuclear genome via endosymbiotic gene transfer (EGT) based on parsimony. *PT* and *Nu* indicate plastid- and nuclear-encoded OGFs, respectively. **c** The total number of gene families that include plastid- and nuclear-encoded OGFs in the three primary plastid groups. *Colored numbers* indicate nuclear-encoded plastid OGFs, for example, among 127 OGFs, 27 EGTs from glaucophytes, 57 EGTs from Viridiplantae, and four EGTs from red algae (see details in Additional file 1: Table S13)

We did an orthologous gene family (OGF) analysis to determine how many plastid genes have been transferred to the nuclear genome via EGT. A total of 297 cyanobacterial orthologous gene clusters were used to represent the set of hypothetical ancestral OGFs. Using 3587 Archaeplastida plastid genes as the query, we found 329,972 cyanobacterial homologs (in 99 strains; Additional file 1: Tables S8 and S9). Using a parsimony approach (Additional file 1: Table S10), the history of gene retention and loss was reconstructed on the Archaeplastida plastid genome phylogeny (see “Methods” and Additional file 8: Figure S23). This analysis showed that 223 OGFs were retained in red algal plastid genomes, whereas 204 OGFs were putatively present in the ancestral plastid genome of the glaucophyte plus green lineage (Fig. 5a). Although similar numbers of OGFs (i.e., 223 versus 204) were retained in the red algal and glaucophyte–green ancestors, these two ancestral lineages shared only 130 OGFs. Glaucophytes (125) and green plants (150) shared 71 plastid OGFs (Fig. 5a). The primordial red algal plastid contained more OGFs [retained 223 (75 %), lost 74] than the glaucophyte [retained 125 (42 %), lost 172] or green ancestor [retained 150 (50 %), lost 147], suggesting extensive differential (and parallel) gene losses after the diversification of each lineage.

To determine if plastid genome reduction was due to outright loss or EGT, we searched for the 297 OGFs in 14 nuclear gene inventories (one glaucophyte, eight green plants, and five red algae; see detail in “Methods”). A total of 956 plastid-derived genes were identified in the nuclear genomes of green plants (886 genes; 76 OGFs, and one plastid OGF-duplicated copy in the nucleus), glaucophytes (48 genes; 33 OGFs), and red algae (22 genes; five OGFs) (Additional file 1: Tables S11 and S12). Among 93 gene families that were lost in the ancestor of the greens and glaucophytes, 23 EGTs were found in both lineages, in addition to five Glaucophyte-specific and 20 Viridiplantae-specific EGTs (Fig. 5b). A total of 79 differential gene family losses followed by five EGTs were identified in the glaucophytes, whereas 54 gene family losses and 33 EGTs were found in Viridiplantae. Therefore, 25 % (76 OGFs) of Viridiplantae and 11 % (33 OGFs) of glaucophyte gene families were transferred to the nuclear genomes in these taxa, whereas only five EGTs (1 %) from 74 lost gene families in red algal plastids were identified as nuclear copies (Fig. 5b). Interestingly, the total number of plastid-associated genes, including those encoded on the plastid genome and those transferred to the nucleus in red algae (228 OGFs) and green plants (226 OGFs) are comparable with 162 (127 + 35 in Fig. 5c) shared OGFs. The three primary plastid lineages share 127 OGFs (Fig. 5c), not only present in plastid genomes but also in nuclear DNA (number of EGT in each lineage = colored number in Fig. 5c; details in Additional file 1: Table S13). These results demonstrate different rates of EGT in Archaeplastida; that is, more frequent in green plants and glaucophytes and less frequent in red algae (Fig. 5 and Additional file 1: Table S11).

To explain the observed patterns of gene conservation and EGT, Barbrook et al. [70] proposed the “limited transfer window hypothesis” based on the finding of higher EGT frequencies in the multiple plastid-containing cells of land plants than in the single plastid-bearing *Chlamydomonas*. These authors hypothesized that EGTs are limited when the host cell converges to a single endosymbiont (plastid). This happened after the integration of cell division genes from lysed endosymbiont DNAs, which were initially multiple in number, because the host cell could not control their division. This reduction in plastid number effectively closed the gene transfer window because lysis of a single plastid would be lethal to the host cell. It is noteworthy that there are significant gene losses (i.e., 93 in glaucophytes plus greens; 79 in the glaucophytes; 54 in green plants; 74 in red alga) and high numbers of EGTs (i.e., 28 out of 33 in the glaucophytes; 43 out of 76 in green plants) during the early evolution of these lineages (Fig. 5a). It is therefore possible that the ancestor of the glaucophyte/green lineage contained multiple plastids, such as *Cyanophora sudae* (two to eight plastids, with generally four per cell [71]), so that frequent EGTs leading to the plastid genome would have been possible. In contrast, most of the early-diverged red algae, including the Cyanidiophyceae, contain a single plastid [72]. This could have suppressed EGTs during their evolution, according to the Barbrook et al. [70] hypothesis.

In contrast, the “nuclear genome reduction hypothesis” [73] provides an alternative explanation for the observation of large red algal plastid genomes. Current data suggest that there was a phase of massive genome reduction in the ancestor of red algae that resulted in 5331–9606 nuclear-encoded proteins in extant taxa with very little EGT from the plastid. The pressure for gene loss in the nucleus could explain the lower success rate of EGT, resulting in a high number of plastid genes (average = 202 proteins) being maintained to support organelle functions. The opposite evolutionary trajectory is observed in the green lineage, which shows expanded nuclear gene inventories (7367–37,109 genes) with high EGT rates (25 %), and, thereby, significant plastid gene loss (average = 84 proteins).

## Conclusions

Analysis of plastid genomes spanning over 1 billion years of Archaeplastida evolution demonstrates that the florideophycean (non-Hildenbrandiophycidae) and seed plant (angiosperm and gymnosperm) lineages have highly conserved genome architectures that have arisen independently in each lineage. This conservation is correlated with the emergence of novel, sexual reproductive structures: carpospore development in Florideophyceae, and seed development in angiosperms and gymnosperms. As a consequence of, or coincident with, these independent rapid radiations, plastid genome architecture was stabilized in the majority of Florideophyceae and seed plant species (Figs. 1 and 3). The carpospore is part of the unique triphasic life cycle (haploid gametophyte, diploid carposporophyte, and diploid tetrasporophyte) that is present in non-hildenbrandiophycidan Florideophyceae. Evolution of the carposporophyte that develops on the female gametophyte led to the production of hundreds of carpospores via post-fertilization development of diploid gonimoblast filaments. This strategy is thought to compensate for inefficient fertilization due to the absence of motile gametes in these attached seaweeds (as in all Rhodophyta) [74]. This innovation likely played a central role in the success and radiation of florideophytes, which account for 95 % of red algal species (Nemaliophycidae 921 spp., Corallinophycidae 772 spp., Ahnfeltiophycidae 11 spp., and Rhodymeniophycidae 5009 spp.; http://www.algaebase.org). Seed plants also exploited novel reproductive strategies to survive in dry (and other stressful) terrestrial conditions. These innovations resulted in seed plants accounting for ~85 % (gymnosperms and angiosperms) of plant species (305,523 species: http://www.theplantlist.org).

Carpospores are estimated to have evolved ~781 mya [54], at the time of the split of Hildenbrandiophycidae from other Florideophyceae. During three periods of global glaciation in the Neoproterozoic era (850–635 mya), the ancestor of Florideophyceae diverged into four red algal subclasses (i.e., 6713 currently recognized species). In contrast, the split time of seed plants is calculated to be ~318 mya [55–57]. Despite the widely different time frames for these events, these complex multicellular and sexually reproducing macrophytes radiated rapidly with the aid of specialized propagation mechanisms (i.e., carpospores and seeds). The explosive diversification of these photosynthetic groups led to highly conserved plastid genome structures in both the red and green plant lineages. Because the plastid is maternally inherited, rapid diversification would lead to conserved organelle genome architecture among closely related taxa. These conserved plastid genomes, however, underwent recombination, aided by duplicated rDNAs or IR regions that resulted in genomic rearrangements. These evolutionary developments could have been adaptive, as found in asexual lineages [75]. In this regard, it has been found that asexuality of scale insects is more common in species with a larger population density and geographic distribution [76]. It is also possible that the evolution of sophisticated retrograde signaling pathways (yet poorly understood in red seaweeds) between the organelle and the nucleus during development [77, 78] could have constrained these conserved plastid genome architectures.

## Methods

### Strain information

Thalli from nine red algal species [*Apophlaea sinclairii* Hooker fils & Harvey, *Ahnfeltia plicata* (Hudson) Fries, *Riquetophycus* sp., *Schimmelmannia schousboei* (J. Agardh) J. Agardh, *Ceramium japonicum* Okamura, *Sebdenia flabellata* (J. Agardh) P. G. Parkinson, *Plocamium cartilagineum* (Linnaeus) P. S. Dixon, *Schizymenia dubyi* (Chauvin ex Duby) J. Agardh, and *Gracilariopsis chorda* (Holmes) Ohmi] were collected from nature and dried with silica-gel. Tissue samples of 11 red algal species [*Porphyridium sordidum* Geitler CCAP 1380, *Bangiopsis subsimplex* (Montagne) F. Schmitz UTEX 2854, *Rhodosorus marinus* Geitler CCMP769, *Rhodella violacea* (Kornmann) Wehrmeyer CCMP 3129, *Rhodella maculata* L. V. Evans CCMP 736, *Erythrotrichia carnea* (Dillwyn) J. Agardh CCMP 3225, *Rhodochaete parvula* Thuret ex Bornet CCMP 3232, *Hildenbrandia rivularis* (Liebmann) J. Agardh UTEX 2622, *Hildenbrandia rubra* (Sommerfelt) Meneghini UTEX 2621, *Asparagopsis taxiformis* (Delile) Trevis. CCAP 1341/1, and *Rhodymenia pseudopalmata* (J. V. Lamour) P. C. Silva UTEX LB1418] were derived from culture collections (Additional file 1: Table S14).

### Genome sequencing, assembly, gene prediction, and annotation

Genomic DNA was extracted from the target species using the DNeasy Plant Mini Kit (Qiagen, Hilden, Germany). NGS was done using the Ion Torrent PGM platform (Life Technologies, San Francisco, CA, USA). The Ion Xpress Plus gDNA Fragment Library Kit (Life Technologies) was used for 200 bp or 400 bp-sized sequencing library preparation. Genome sequencing was done with the Ion PGM Template OT2 200 or 400 Kit and Ion PGM Sequencing 200 or 400 Kit (Life Technologies). The raw sequence reads were assembled using the CLC Genomics Workbench 5.5.1 (CLC Bio, Aarhus, Denmark) and the MIRA assembler (from Ion Server). Plastid genome-related contigs were sorted by customized Python scripts with local BLAST programs compared with references, and the sorted contigs were re-assembled to construct consensus plastid genomes. Initial consensus plastid genomes were confirmed with the read-mapping method using CLC Genomics Workbench 5.5.1 and gaps were filled with PCR.

Putative ORFs in the plastid genome data were predicted using ORF Finder in Geneious 8.1.2 [79], customized Python scripts, and BLASTx tool (*e*-value ≤1.0*e* ^−05^) with codon table 11 (Bacterial, Archaeal, and Plant Plastid Code). Ribosomal DNAs and transfer RNAs were predicted using the RNAmmer 1.2 Server [80], and the ARAGORN program [81]. All introns were identified using the web-based program RNAweasel (http://www.theplantlist.org) [82–86].

### Gene clustering of orthologous plastid gene families and phylogenetic analysis

All 48 available red algal plastid genome data were used to construct OGFs, excluding the parasitic species (*Choreocolax polysiphoniae*) that contained a highly reduced collection of plastid gene families [6]. All individual genes in 48 red algal species were used in a three-step gene clustering procedure with customized Python scripts with the local BLASTp program. In the first step the script collected OGFs in all other species compared with individual genes of a specified species (*e*-value ≤ *e* ^−10^). Second, the script re-collected gene families from unrecognized species in the first step using already collected gene families. Finally, non-orthologous genes (but very closely related with high similarity) were checked manually and eliminated from each grouped gene family. Red algal plastid *rpo*C1 and *rpo*C2 genes were combined as one fragment for their alignment because the plastid genomes of *Cyanidioschyzon merolae* and Cyanidiales sp. encode a combined *rpo*C gene. After several iterations, 191 red algal plastid gene families were chosen for analysis (Additional file 1: Table S4). This clustering method was adapted to the green lineage and 77 green plastid gene families were chosen (Additional file 1: Table S7).

The 60 conserved plastid genes from the three primary plastid groups and cyanobacterial homologs were used to reconstruct a ML phylogeny (Additional file 1: Tables S15 and S16). This tree topology provided the framework for the analysis of primary plastid gene retention, loss, or transfer to the nucleus (Additional file 8: Figure S23). One difference between the primary plastid phylogeny and the green plant-specific ML tree (Figs. 1 and 3) was the position of the monophyletic charophyte algae *Mesostigma* and *Chlorokybus* that was located basal to green plants. This incongruence has been previously discussed [87].

All intronic ORFs of the *trn*Me tRNA in the Florideophyceae were grouped with their homologs (top match 30 genes), and searched by BLASTp (*e*-value ≤ *e* ^−05^) using the nr database (NCBI) and our local database (red algal plastid genes). The collected plastid coding genes were used for multigene phylogenetic analysis. Each clustered gene set was aligned using MAFFT 7.110 under default settings [88]. All of the alignments were concatenated for the phylogenetic analysis. To construct the tree, phylogenetic models were tested (-m TEST), and the ML tree search and their bootstrap analysis were done using the IQ tree program with 1000 replications (-bb 1000) [89–91].

To test the inferred tree topology, we conducted the tree topology test using CONSEL [41]. We first built a ML tree excluding R3-type species with 1000 bootstrap replicates with IQ tree. Based on this ML tree, we tested the hypothetical tree topology that R1- and R2-types form monophyletic groups, and excluded the R3-type. From CONSEL, the result was generated using 10,000 bootstrap replicates. Statistical support was calculated with the AU test [42].

### Comparison of structural variation in plastid genomes

To compare plastid genome architectures from a taxonomically broadly sampled collection of taxa, all available plastid genomes in the red and green lineages were aligned and plotted using the MUMmerplot package [40]. Excluding a parasitic red algal plastid genome, the structural variation of 45 red algal complete plastid genomes was analyzed. A total of 869 green plastid genomes were used for this analysis. In the 703 angiosperm plastid genomes, similar plastid genome architectures were categorized into three major three (A1–A3-type) and the remainder were put in Rest-type based on the plotting result (Additional file 4: Figures S11–S14). The A1-type was the most common type of angiosperm plastid genome. Except highly diverse plastid genome architectures contained green algae (58 spp.) and charophytes (11 spp.), four major representative types of plastid genomes in other groups [gymnosperms (67 spp.), pteridophytes (18 spp.), bryophytes (12 spp.), and charophytes (11 spp.)] were chosen for the futher structural and phylogenetic analyses (Additional file 4: Figures S7–S10). Structural comparison of plastid genomes was done using MAUVE 2.3.1 [92] under “default options.” Synteny of plastid genes was assessed manually based on the results of the MAUVE alignment. BDs were calculated based on MAUVE comparison using the DCJ analysis tool in the Geneious 8.1.2 [79].

### Orthologous gene family analysis

A total of 329,972 genes were sampled from 99 cyanobacterial strains (NCBI genome database) (Additional file 1: Table S8). A total of 81,476 primary plastid genes (149 genes from one glaucophyte, 71,558 genes from 868 green plants, and 9739 genes from 48 red algae; Additional file 1: Tables S17 and S18) were searched using BLASTp (*e*-value ≤1.0*e* ^−05^) against the local cyanobacterial database. An analysis using customized Python scripts with a BLASTp search (*e*-value 1.0*e* ^−10^) (Additional file 1: Table S9) found that a total of 297 OGFs clustered from 3587 cyanobacterial homologs were shared between cyanobacteria and primary plastid genomes, but 2568 sequences were of an unknown-origin. Based on the 297 OGFs including 78,908 sequences, a parsimonious evolutionary scheme for gene families was calculated (Fig. 5 and Additional file 1: Table S10). The OGF total of 80,849 primary plastid genes was predicted using BLASTp (*e*-value ≤ 1.0*e* ^−05^) based on the ancestral 297 OGFs and the classified plastid genes that were used to find secondary (indirect) relationships from non-predicted primary plastid genes using a BLASTp search (*e*-value ≤ *e* ^−10^). From a total of 78,908 primary plastid genes including 2568 indirect relationships genes, OGF groups were predicted. Parsimonious gene family evolution was analyzed and the result was included in the simplified tree (Fig. 5 and Additional file 1: Table S10). For instance, 149 plastid genes in the glaucophyte *Cyanophora paradoxa* included 143 cyanobacterial homologs that clustered into 125 OGFs, and six of unknown or non-EGT origin (Fig. 5a).

To identify cases of EGT from the plastid to the nuclear genome, 14 nuclear genomes from the primary endosymbiosis groups (one glaucophyte, eight green plants, and five red algae; Additional file 1: Table S19) were used for a local BLASTp search (*e*-value ≤1.0*e* ^−05^) against 297 OGF sequences. Putative plastid-derived, nuclear-encoded genes were combined with the hypothetical OGF evolutionary tree (Fig. 5). The collected genes were each used in a BLASTp search (*e*-value ≤1.0*e* ^−05^) against our local RefSeq database. From these BLASTp results, the top five hits in each taxonomic group were combined and aligned using MAFFT 7.110 under default settings [88]. All of these alignments were analyzed using IQ tree (model test: -m TEST and replications: -bb 1000) [89–91] and used only the cyanobacterial-origin ML tree to plot EGT information (Fig. 5b, c).

## Additional files

Additional file 1: Table S1. Gene compositions of red algal plastid genomes. **Table S2.** Representative homologous gene sets of red algal plastid genes. **Table S3.** Intron prediction of plastid intronic proteins in five basal red algal taxa. **Tables S4, S7, and S15.** List of concatenated proteins used in the ML tree searches. **Tables S5 and S6.** Types of angiosperm and gymnosperm plastid genomes. **Tables S8 and S9.** Taxon sampling of cyanobacterial genomes and their gene groups. **Table S10.** Predicted primary plastid orthologous gene families compared with cyanobacterial 297 OGF. **Table S11.** Predicted nuclear-encoded OGFs in the primary plastid groups. **Table S12.** EGT trees information. **Table S13.** Composition of OGFs in the primary plastid groups. **Table S14.** Sample and strain information. **Table S16.** Cyanobacterial proteins from four strains used in the primary plastid ML tree. **Table S17.** Taxon sampling of primary plastid groups in orthologous family analysis. **Table S18.** Number of species and genes in OGF analysis. **Table S19.** Taxon samples and their genes in the primary endosymbiosis groups. (XLSX 2166 kb)
Additional file 2: Figure S1. ML tree made using homologous genes based on intronic ORF of *trn*Me tRNA in Florideophyceae. (PDF 131 kb)
Additional file 3: Figure S2. ML tree built using aligned 191 concatenated proteins from 48 red algal plastid genomes. **Figure S3.** Structural comparison of red algal plastid genomes based on MUMmerplot result. **Figure S4.** Structural comparison of plastid genomes from four Cyanidiophyceae species based on MAUVE alignment result. **Figure S5.** Structural comparison of plastid genomes in three basal red algal groups. (PDF 1955 kb)
Additional file 4: Figure S6. Structural comparison of green lineage plastid genomes based on MUMmerplot result. **Figures S7–S15.** Types of green lineage (angiosperm, gymnosperm, bryophytes, pteridophytes, green algae, charophytes and volvocine algae) plastid genomes from MUMmerplot. (PDF 1201 kb)
Additional file 5: Figure S16. Structural comparison of Geraniales plastid genomes based on MUMmerplot. (PDF 198 kb)
Additional file 6: Figure S17. Structural comparison of brown lineage plastid genomes based on MUMmerplot. **Figure S18.** Structural comparison of cryptophyte plastid genomes based on MUMmerplot. **Figure S19.** Structural comparison of diatom lineage plastid genomes based on MUMmerplot. **Figure S20.** Structural comparison of Eustigmatophyceae plastid genomes based on MUMmerplot. **Figure S21.** Structural comparison of haptophytes plastid genomes based on MUMmerplot. (PDF 503 kb)
Additional file 7: Figure S22. Box plots (standard deviation) of 770 seed plants and 24 red algal plastid genomes. (PDF 70 kb)
Additional file 8: Figure S23. ML tree based on concatenated 60 plastid genes from Archaeplastida and their cyanobacterial homologs. (PDF 106 kb)

## Abbreviations

AU: Approximately unbiased
BD: Breakpoint distance
CDS: Coding sequences
EGT: Endosymbiotic gene transfer
IR: Inverted repeat
LSC: Large single copy
ML: Maximum likelihood
MLB: Maximum likelihood bootstrap support
mya: Million years ago
NGS: Next-generation sequencing
OGF: Orthologous gene family
ORF: Open-reading frame
rDNA: Ribosomal DNA operon
SSC: Small single copy
tRNA: Transfer RNA

## References

[1] Bhattacharya D, Yoon HS, Hackett JD (2004). Photosynthetic eukaryotes unite: endosymbiosis connects the dots. BioEssays.

[2] Cavalier-Smith T (2000). Membrane heredity and early chloroplast evolution. Trends Plant Sci.

[3] Rodríguez-Ezpeleta N, Brinkmann H, Burey SC, Roure B, Burger G, Löffelhardt W (2005). Monophyly of primary photosynthetic eukaryotes: green plants, red algae, and glaucophytes. Curr Biol.

[4] Larkum AW, Lockhart PJ, Howe CJ (2007). Shopping for plastids. Trends Plant Sci.

[5] Muller HJ (1932). Some genetic aspects of sex. Am Nat.

[6] Salomaki ED, Nickles KR, Lane CE (2015). The ghost plastid of *Choreocolax polysiphoniae*. J Phycol.

[7] Schelkunov MI, Shtratnikova VY, Nuraliev MS, Selosse MA, Penin AA, Logacheva MD (2015). Exploring the limits for reduction of plastid genomes: a case study of the mycoheterotrophic orchids *Epipogium aphyllum* and *Epipogium roseum*. Genome Biol Evol.

[8] Martin W, Rujan T, Richly E, Hansen A, Cornelsen S, Lins T (2002). Evolutionary analysis of *Arabidopsis*, cyanobacterial, and chloroplast genomes reveals plastid phylogeny and thousands of cyanobacterial genes in the nucleus. Proc Natl Acad Sci U S A.

[9] Timmis JN, Ayliffe MA, Huang CY, Martin W (2004). Endosymbiotic gene transfer: organelle genomes forge eukaryotic chromosomes. Genetics.

[10] Richardson AO, Palmer JD (2007). Horizontal gene transfer in plants. J Exp Bot.

[11] Keeling PJ, Palmer JD (2008). Horizontal gene transfer in eukaryotic evolution. Nat Rev Genet.

[12] Keeling PJ (2004). Diversity and evolutionary history of plastids and their hosts. Am J Bot.

[13] Keeling PJ, Burget G, Durnford DG, Lang BF, Lee RW, Pearlman RE (2005). The tree of eukaryotes. Trends Ecol Evol.

[14] Keeling PJ (2010). The endosymbiotic origin, diversification and fate of plastids. Phil Trans R Soc B.

[15] Reith M, Munholland J (1995). Complete nucleotide sequence of the *Porphyra purpurea* chloroplast genome. Plant Mol Biol Rep.

[16] Glöckner G, Rosenthal A, Valentin K (2000). The structure and gene repertoire of an ancient red algal plastid genome. J Mol Evol.

[17] Ohta N, Matsuzaki M, Misumi O, Miyagishima S, Nozaki H, Tanaka K (2003). Complete sequence and analysis of the plastid genome of the unicellular red alga *Cyanidioschyzon merolae*. DNA Res.

[18] Hagopian JC, Reis M, Kitajima JP, Bhattacharya D, de Oliveira MC (2004). Comparative analysis of the complete plastid genome sequence of the red alga *Gracilaria tenuistipitata* var. *liui* provides insights into the evolution of rhodoplasts and their relationship to other plastid. J Mol Evol.

[19] Collén J, Porcel B, Carré W, Ball SG, Chaparro C, Tonon T (2013). Genome structure and metabolic features in the red seaweed *Chondrus crispus* shed light on evolution of the Archaeplastida. Proc Natl Acad Sci U S A.

[20] DePriest MS, Bhattacharya D, López-Bautista JM (2013). The plastid genome of the red macroalga *Grateloupia taiwanensis* (Halymeniaceae). PLoS One.

[21] Janouškovec J, Liu SL, Martone PT, Carré W, Leblanc C, Collén J (2013). Evolution of red algal plastid genome: ancient architectures, introns, horizontal gene transfer, and taxonomic utility of plastid markers. PLoS One.

[22] Wang L, Mao Y, Kong F, Li G, Ma F, Zhang B (2013). Complete sequence and analysis of plastid genomes of two economically important red algae: *Pyropia haitanensis* and *Pyropia yezoensis*. PLoS One.

[23] Campbell MA, Presting G, Bennett MS, Sherwood AR (2014). Highly conserved organellar genomes in the Gracilariales as inferred using new data from the Hawaiian invasive alga *Gracilaria salicornia* (Rhodophyta). Phycologia.

[24] Hughey JR, Gabrielson PW, Tohmer L, Tortolani J, Silva M, Miller KA (2014). Minimally destructive sampling of type specimens of *Pyropia* (Bangiales, Rhodophyta) recovers complete plastid and mitochondrial genomes. Sci Rep..

[25] Tajima N, Sato S, Maruyama F, Kurokawa K, Ohta H, Tabata S (2014). Analysis of the complete plastid genome of the unicellular red alga *Porphyridium purpureum*. J Plant Res.

[26] Jain K, Krause K, Grewe F, Nelson GF, Weber APM, Chriatenaen AC (2015). Extreme features of the *Galdieria sulphuraria* organellar genomes: a consequence of polyextremophily?. Genome Biol Evol.

[27] Lee JM, Kim KM, Yang EC, Miller KA, Boo SM, Bhattacharya D (2016). Reconstructing the complex evolutionary history of mobile plasmids in red algal genomes. Sci Rep..

[28] Lemieux C, Otis C, Turmel M (2014). Chloroplast phylogenomic analysis resolves deep-level relationships within the green algal class Trebouxiophyceae. BMC Evol Biol.

[29] Ruhfel BR, Gitzendanner MA, Soltis PS, Solits DE, Burleigh JG (2014). From algae to angiosperms-inferring the phylogeny of green plants (Viridiplantae) from 360 plastid genomes. BMC Evol Biol.

[30] Leliaert F, Tronholm A, Lemieux C, Turmel M, DePriest MS, Bhattacharya D (2016). Chloroplast phylogenomic analyses reveal the deepest-branching lineage of the Chlorophyta, Palmophyllophyceae class. nov. Sci Rep..

[31] Palmer JD, Delwiche CF, Soltis DE, Soltis PS, Doyle JJ (1998). The origin and evolution of plastids and their genomes. Molecular systematic of plants II: DNA sequencing.

[32] Bancroft I, Wolk CP, Oren EV (1989). Physical and genetic maps of the genome of the heterocyst-forming cyanobacterium *Anabaena* sp. strain PCC7120. J Bacteriol.

[33] Chen X, Widger WR (1993). Physical genome map of the unicellular cyanobacterium *Synechococcus* sp. strain PCC7002. J Bacteriol.

[34] Kaneko T, Sato S, Kotani H, Tanaka A, Asamizu E, Nakamura Y (1996). Sequence analysis of the genome of the unicellular cyanobacterium *Synechocystis* sp. strain PCC6803. II. Sequence determination of the entire genome and assignment of potential protein-coding regions. DNA Res.

[35] Yoon HS, Müller KM, Sheath RG, Ott FD, Bhattacharya D (2006). Defining the major lineages of red algae (Rhodophyta). J Phycol.

[36] Le Gall L, Saunders GW (2007). A nuclear phylogeny of the Florideophyceae (Rhodophyta) inferred from combined EF2, small subunit and large subunit ribosomal DNA: establishing the new red algal subclass Corallinophycidae. Mol Phylogenet Evol.

[37] Verbruggen H, Maggs CA, Saunders GW, Le Gall L, Yoon HS (2010). Data mining approach identifies research priorities and data requirements for resolving the red algal tree of life. BMC Evol Biol..

[38] Yoon HS, Hackett JD, Bhattacharya D (2002). A single origin of the peridinin- and fucoxanthin-containing plastids in dinoflagellates through tertiary endosymbiosis. Proc Natl Acad Sci U S A.

[39] Yoon HS, Hackett JD, Ciniglia C, Pinto G, Bhattacharya D (2004). A molecular timeline for the origin of photosynthetic eukaryotes. Mol Biol Evol.

[40] Kurtz S, Phillippy A, Delcher AL, Smoot M, Shumway M, Antonescu C (2004). Versatile and open software for comparing large genomes. Genome Biol..

[41] Shimodaira H, Hasegawa M (2001). CONSEL: for assessing the confidence of phylogenetic tree selection. Bioinformatics.

[42] Shimodaira H (2002). An approximately unbiased test of phylogenetic tree selection. Syst Biol.

[43] Muller HJ (1964). The relation of recombination to mutational advance. Mut Red.

[44] Palmer JD (1983). Chloroplast DNA, exists in two orientations. Nature..

[45] Palmer JD (1985). Comparative organization of chloroplast genomes. Annu Rev Genet.

[46] Aldrich J, Cherney B, Merlin E, Williams C, Mets L (1985). Recombination within the inverted repeat sequences of the *Chlamydomonas reinhardtii* chloroplast genome produces 2 orientation isomers. Curr Genet.

[47] Stein DB, Palmer JD, Thompson WF (1986). Structural evolution and flip-flop recombination of chloroplast DNA in the fern genus *Osmunda*. Curr Genet.

[48] Palmer JD, Osorio B, Aldrich J, Thompson WF (1987). Chloroplast DNA evolution among legumes: loss of a large inverted repeat occurred prior to other sequences rearrangements. Curr Genet.

[49] Bourne CM, Palmer JD, Stoermer EF (1992). Organization of the chloroplast genome of the freshwater centric diatom *Cyclotella meneghiniana*. J Phycol.

[50] Linne von Berg KH, Kowallik KV (1992). Structural organization of the chloroplast genome of the chromophytic alga *Vaucheria bursata*. Plant Mol Biol.

[51] Cattolico RA, Jacobs MA, Zhou Y, Chang J, Duplessis M, Lybrand T (2008). Chloroplast genome sequencing analysis of *Heterosigma akashiwo* CCMP452 (West Atlantic) and NIES293 (West Pacific) strains. BMC Genomics.

[52] Walker JF, Jansen RK, Zanis MJ, Emery NC (2015). Sources of inversion variation in the small single copy (SSC) region of chloroplast genomes. Am J Bot.

[53] Zhu A, Guo W, Gupta S, Fan W, Mower JP (2015). Evolutionary dynamics of the plastid inverted repeat: the effects of expansion, contraction, and loss on substitution rates. New Phytol..

[54] Yang EC, Boo SM, Bhattacharya D, Saunders GW, Knoll AH, Fredericq S (2016). Divergence time estimates and the evolution of major lineages in the florideophyte red algae. Sci Rep..

[55] Rothwell GW, Scheckler SE, Gillespie WH (1989). *Elkinsia* gen. nov., a late Devonian gymnosperm with cupulate ovules. Bot Gaz.

[56] Smith SA, Beaulieu JM, Donoghue MJ (2010). An uncorrelated relaxed-clock analysis suggests an earlier origin for flowering plants. Proc Natl Acad Sci U S A.

[57] Magallón S, Hilu KW, Quandt D (2013). Land plant evolutionary timeline: gene effects are secondary to fossil constraints in relaxed clock estimation of age and substitution rates. Am J Bot.

[58] Maréchal A, Parent J, Véronneau-Lafortune F, Joyeux A, Lang BF, Brisson N (2009). Whirly proteins maintain plastid genome stability in *Arabidopsis*. Proc Natl Acad Sci U S A.

[59] Hu J, Wang D, Li J, Jing G, Ning K, Xu J (2014). Genome-wide identification of transcription factors and transcription-factor binding sites in oleaginous microalgae *Nannochloropsis*. Sci Rep..

[60] Guisinger MM, Kuehl JV, Boore JL, Jansen RK (2011). Extreme reconfiguration of plastid genomes in the angiosperm family Geraniaceae: rearrangements, repeats, and codon usage. Mol Biol Evol.

[61] Weng ML, Blazier JC, Govindu M, Jansen RK (2013). Reconstruction of the ancestral plastid genome in Geraniaceae reveals a correlation between genome rearrangements, repeats, and nucleotide substitution rates. Mol Biol Evol.

[62] Zhang J, Ruhlman TA, Sabir JSM, Blazier JC, Weng M, Park S (2016). Coevolution between nuclear-encoded DNA replication, recombination, and repair genes and plastid genome complexity. Genome Biol Evol.

[63] Silberfeld T, Leigh JW, Verbruggen H, Cruaud C, de Reviers B, Rousseau F (2010). A multi-locus time-calibrated phylogeny of the brown algae (Heterokonta, Ochrophyta, Phaeophyceae): Investigating the evolutionary nature of the “brown algal crown radiation”. Mol Phylogenet Evol..

[64] Liu F, Pang S (2016). Chloroplast genome of *Sargassum horneri* (Sargassaceae, Phaeophyceae): comparative chloroplast genomics of brown algae. J Appl Phycol.

[65] Yang JH, Graf L, Cho CH, Jeon BH, Kim JH, Yoon HS (2016). Complete plastid genome of an ecologically important brown alga *Sargassum thunbergii* (Fucales, Phaeophyceae). Mar Genomics..

[66] Chumley TW, Palmer JD, Mower JP, Fourcade HM, Calie PJ, Boore JL (2006). The complete chloroplast genome sequence of *Pelargonium x hortorum*: organization and evolution of the largest and most highly rearranged chloroplast genome of land plants. Mol Biol Evol.

[67] Nei M, Gojobori T (1986). Simple methods for estimating the numbers of synonymous and nonsynonymous nucleotide substitutions. Mol Biol Evol.

[68] Martin W, Stoebe B, Goremykin V, Hansmann S, Hasegawa M, Kowallik KV (1998). Gene transfer to the nucleus and the evolution of chloroplasts. Nature.

[69] Reyes-Prieto A, Yoon HS, Moustafa A, Yang EC, Andersen RA, Boo SM (2010). Differential gene retention in plastids of common recent origin. Mol Biol Evol.

[70] Barbrook AC, Howe CH, Purton S (2006). Why are plastid genomes retained in non-photosynthetic organisms?. Trends Plant Sci.

[71] Takahashi T, Sato M, Toyooka K, Matsuzaki R, Kawafune K, Kawamura M (2014). Five *Cyanophora* (Cyanophorales, Glaucophyta) species delineated based on morphological and molecular data. J Phycol.

[72] Pinto G, Albertano P, Ciniglia C, Cozzolino S, Pollio A, Yoon HS (2003). Comparative approaches to the taxonomy of the genus *Galdieria merola* (Cyanidiales, Rhodophyta). Cryptogam Algol.

[73] Qiu H, Price DC, Yang EC, Yoon HS, Bhattacharya D (2015). Evidence of ancient genome reduction in red algae (Rhodophyta). J Phycol.

[74] Searles RB (1980). The strategy of the red algal life history. Amer Nat.

[75] Seidl MF, Thomma BPHJ (2014). Sex or no sex: evolutionary adaptation occurs regardless. Bioessays.

[76] Ross L, Hardy NB, Okusu A, Normark BB (2012). Large population size predicts the distribution of asexuality in scale insects. Evolution.

[77] Gollan PJ, Tikkanen M, Aro EM (2015). Photosynthetic light reactions: integral to chloroplast retrograde signalling. Curr Opin Plant Biol..

[78] Tadini L, Pesaresi P, Kleine T, Rossi F, Guljamow A, Sommer F (2016). GUN1 controls accumulation of the plastid ribosomal protein S1 at the protein level and interacts with proteins involved in plastid protein homeostasis. Plant Physiol.

[79] Kearse M, Moir R, Wilson A, Stones-Hava S, Cheung M, Sturrock S (2012). Geneious Basic: an integrated and extendable desktop software platform for the organization and analysis of sequence data. Bioinformatics.

[80] Lagesen K, Hallin P, Rødland EA, Stærfeldt HH, Rognes T, Ussery DW (2007). RNAmmer: consistent and rapid annotation of ribosomal RNA genes. Nucleic Acids Res.

[81] Laslett D, Canback B (2004). ARAGORN, a program to detect tRNA genes and tmRNA genes in nucleotide sequences. Nucleic Acids Res.

[82] Eddy SR, Durbin R (1994). RNA sequence analysis using covariance models. Nucleic Acids Res.

[83] Smith SW, Overbeek R, Woese CR, Gilbert W, Gillevet PM (1994). The genetic data environment an expandable GUI for multiple sequence analysis. Comput Appl Biosci.

[84] Gautheret D, Lambert A (2001). Direct RNA motif definition and identification from multiple sequence alignments using secondary structure profiles. J Mol Biol.

[85] Lang BF, Laforest MJ, Burger G (2007). Mitochondrial introns: a critical view. Trends Genet.

[86] Burger G, Yan Y, Javadi P, Lang BF (2009). Group I-intron trans-splicing and mRNA editing in mitochondria of placozoan animals. Trends Genet.

[87] Lemieux C, Otis C, Turmel M (2007). A clade uniting the green algae *Mesostigma viride* and *Chlorokybus atmophyticus* represents the deepest branch of the Streptophyta in chloroplast genome-based phylogenies. BMC Biol.

[88] Katoh K, Toh H (2008). Recent developments in the MAFFT multiple sequence alignment program. Brief Bioinform.

[89] Minh BQ, Nguyen MAT, Von Haeseler A (2013). Ultrafast approximation for phylogenetic bootstrap. Mol Biol Evol.

[90] Flouri T, Izquierdo-Carrasco F, Darriba D, Aberer AJ, Nguyen LT, Minh BQ (2015). The phylogenetic likelihood library. Syst Biol.

[91] Nguyen LT, Schmidt HA, Von Haeseler A, Minh BQ (2015). IQ-TREE: A fast and effective stochastic algorithm for estimating maximum-likelihood phylogenies. Mol Biol Evol.

[92] Darling ACE, Mau B, Blattner FR, Perna NT (2004). Mauve: multiple alignment of conserved genomic sequence with rearrangements. Genome Res.

