# Supplementary material for: Parallel evolution of highly conserved plastid genome architecture in red seaweeds and seed plants
Source: BMC Biol. 2016 Sep 2;14:75. doi: 10.1186/s12915-016-0299-5 (PMC5010701; doi:10.1186/s12915-016-0299-5)
Supplement: Additional file 2: Figure S1. — ML tree made using homologous genes based on intronic ORF of trnMe tRNA in Florideophyceae. (PDF 131 kb) [file 12915_2016_299_MOESM2_ESM.pdf]

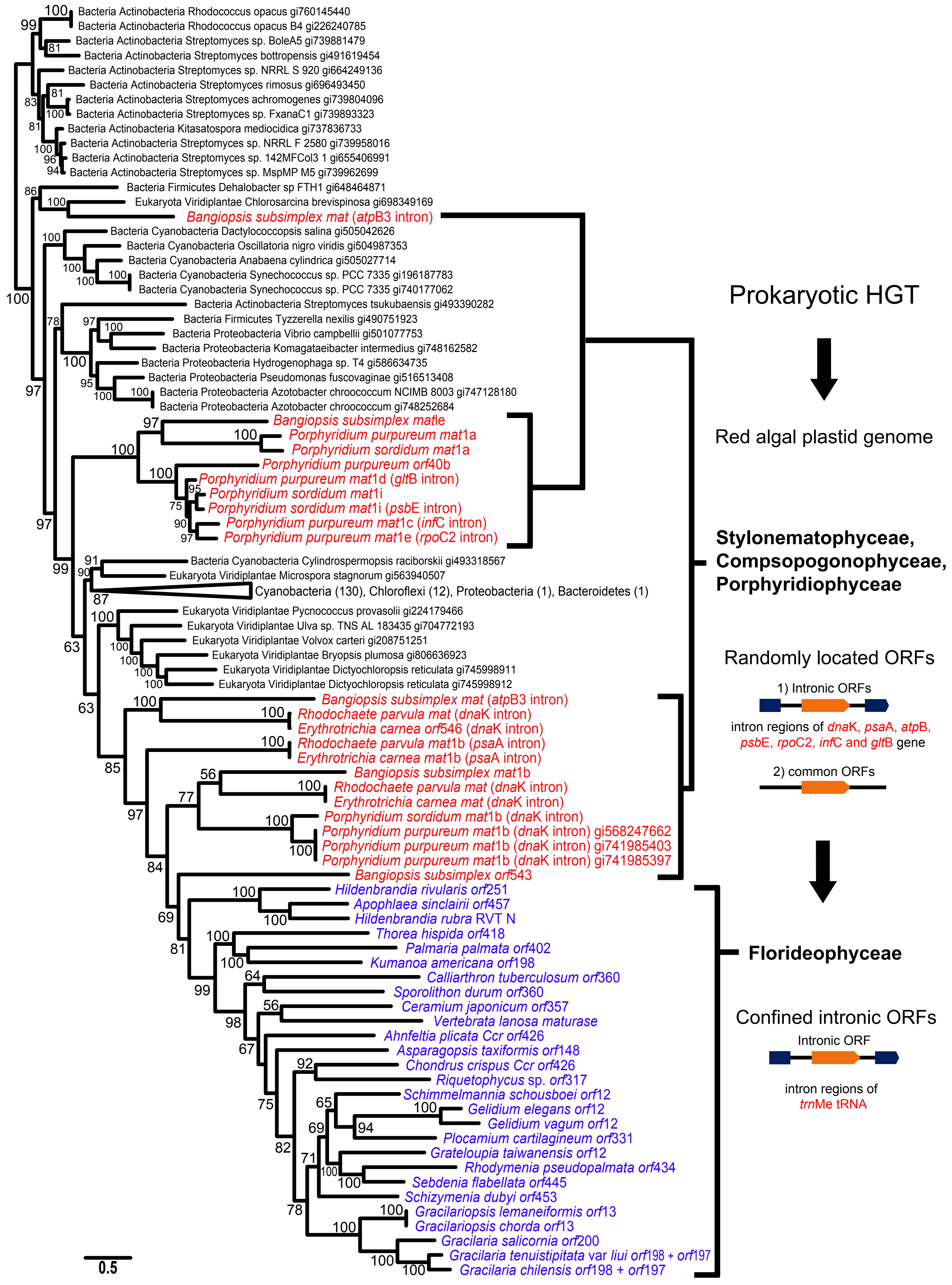

Figure S1. ML tree made using homologous genes based on intronic ORF of *trn*Me tRNA in Florideophyceae (1000 replications, bootstrap support when > 50%). Species in red text indicate the Stylonematophyceae, Compsopogonophyceae and Porphyridiophyceae species that the homologous proteins are located in intron regions of *dna*K, *psa*A, *atp*B, *psb*E, *rpo*C2, *inf*C and *glt*B gene as well as non-coding region (common ORFs). Blue colored species names indicate the Florideophyceae species that proteins are located in intron region of *trn*Me tRNA.
