# Supplementary material for: Parallel evolution of highly conserved plastid genome architecture in red seaweeds and seed plants
Source: BMC Biol. 2016 Sep 2;14:75. doi: 10.1186/s12915-016-0299-5 (PMC5010701; doi:10.1186/s12915-016-0299-5)
Supplement: Additional file 3: Figure S2. — ML tree built using aligned 191 concatenated proteins from 48 red algal plastid genomes. Figure S3. Structural comparison of red algal plastid genomes based on MUMmerplot result. Figure S4. Structural comparison of plastid genomes from four Cyanidiophyceae species based on MAUVE alignment result. Figure S5. Structural comparison of plastid genomes in three basal red algal groups. (PDF 1955 kb) [file 12915_2016_299_MOESM3_ESM.pdf]

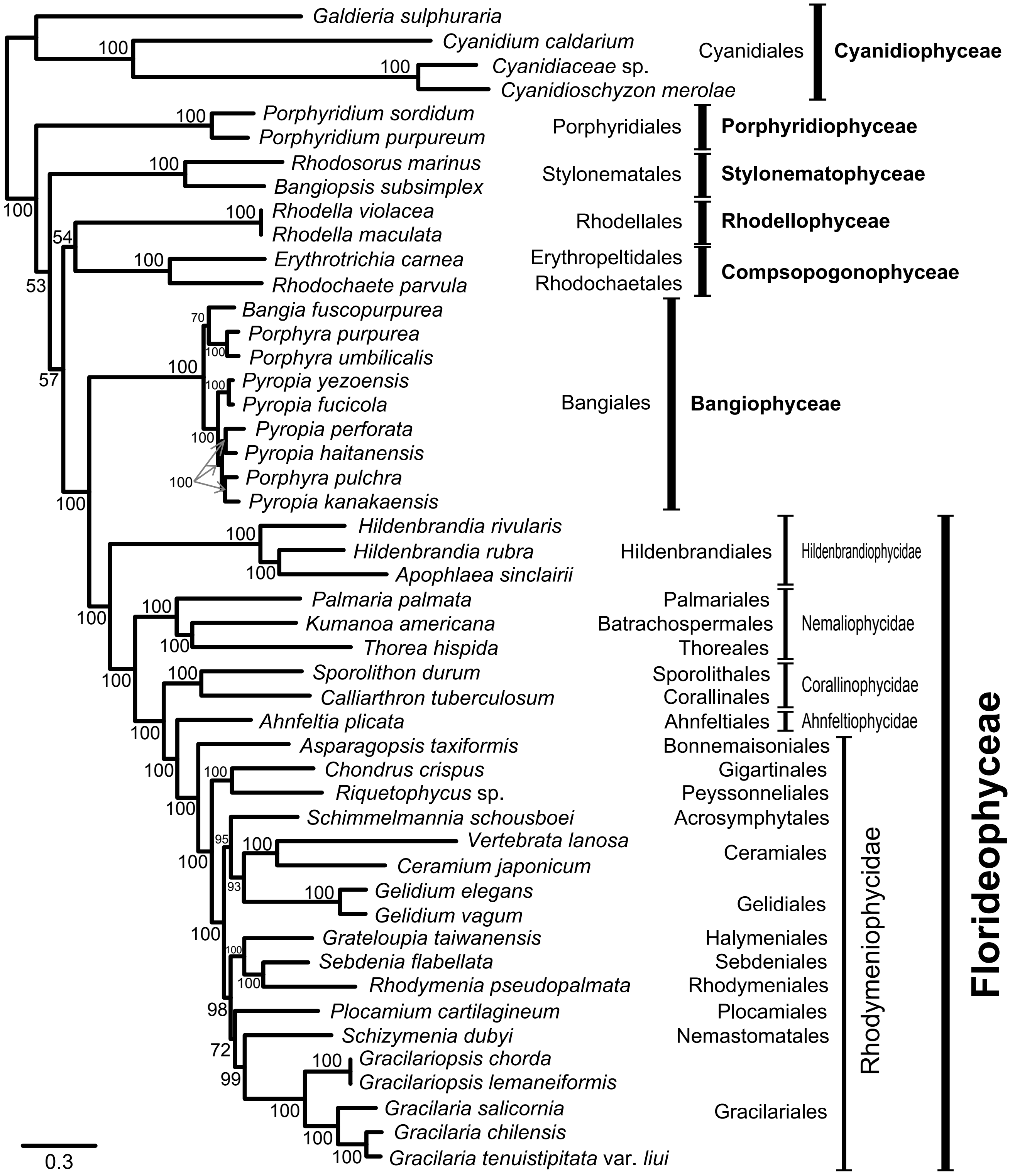

Figure S2. ML tree built using aligned 191 concatenated proteins from 48 red algal plastid genomes (1000 replications, bootstrap support when > 50%).

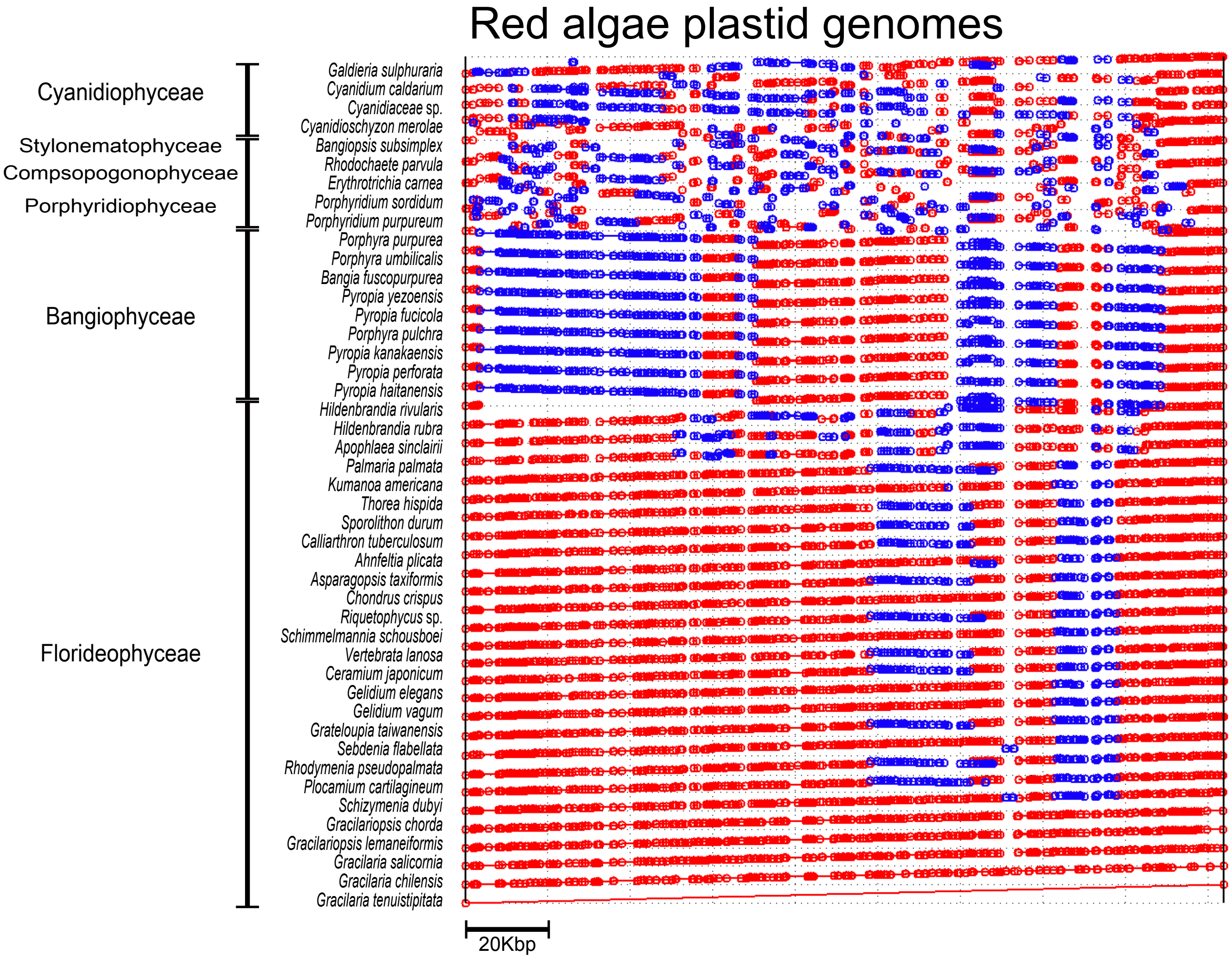

Figure S3. Structural comparison of red algal plastid genomes based on MUMmerplot result. All plastid genome architectures are compared with the plastid genome of *Gracilaria tenuistipitata* as recent diverged species.

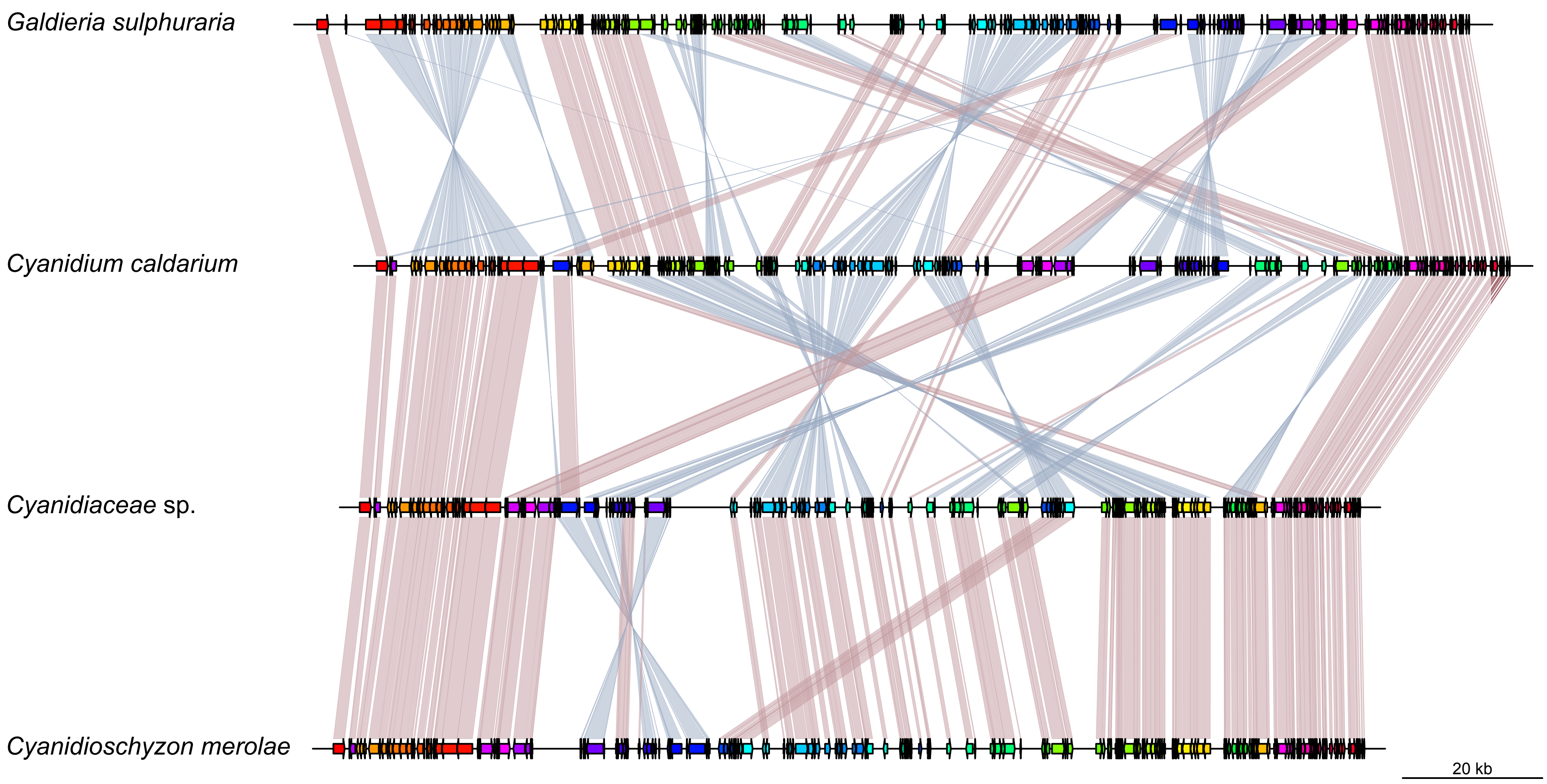

Figure S4. Structural comparison of plastid genomes from four Cyanidiophyceae species based on MAUVE alignment result.

Red algal 3 basal groups plastid genomes

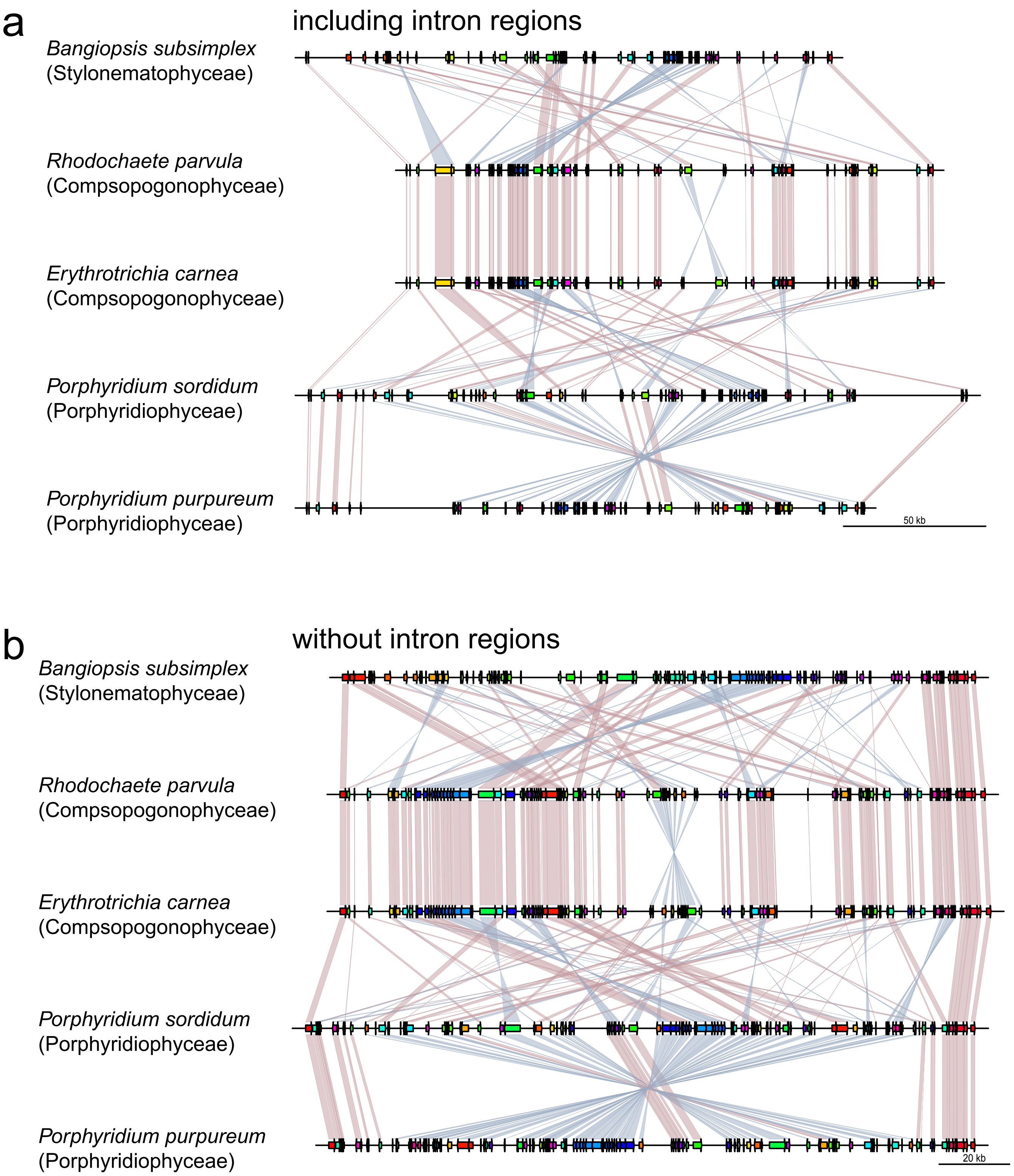

Figure S5. Structural comparison of plastid genomes in red algal 3 basal groups. Including intron regions ‘a’ and without intron regions ‘b’ are shown from the class Stylonematophyceae, Compsopogonophyceae and Porphyridiophyceae.
