# Supplementary material for: Parallel evolution of highly conserved plastid genome architecture in red seaweeds and seed plants
Source: BMC Biol. 2016 Sep 2;14:75. doi: 10.1186/s12915-016-0299-5 (PMC5010701; doi:10.1186/s12915-016-0299-5)
Supplement: Additional file 4: Figure S6. — Structural comparison of green lineage plastid genomes based on MUMmerplot result. Figures S7–S15. Types of green lineage (angiosperm, gymnosperm, bryophytes, pteridophytes, green algae, charophytes and volvocine algae) plastid genomes from MUMmerplot. (PDF 1201 kb) [file 12915_2016_299_MOESM4_ESM.pdf]

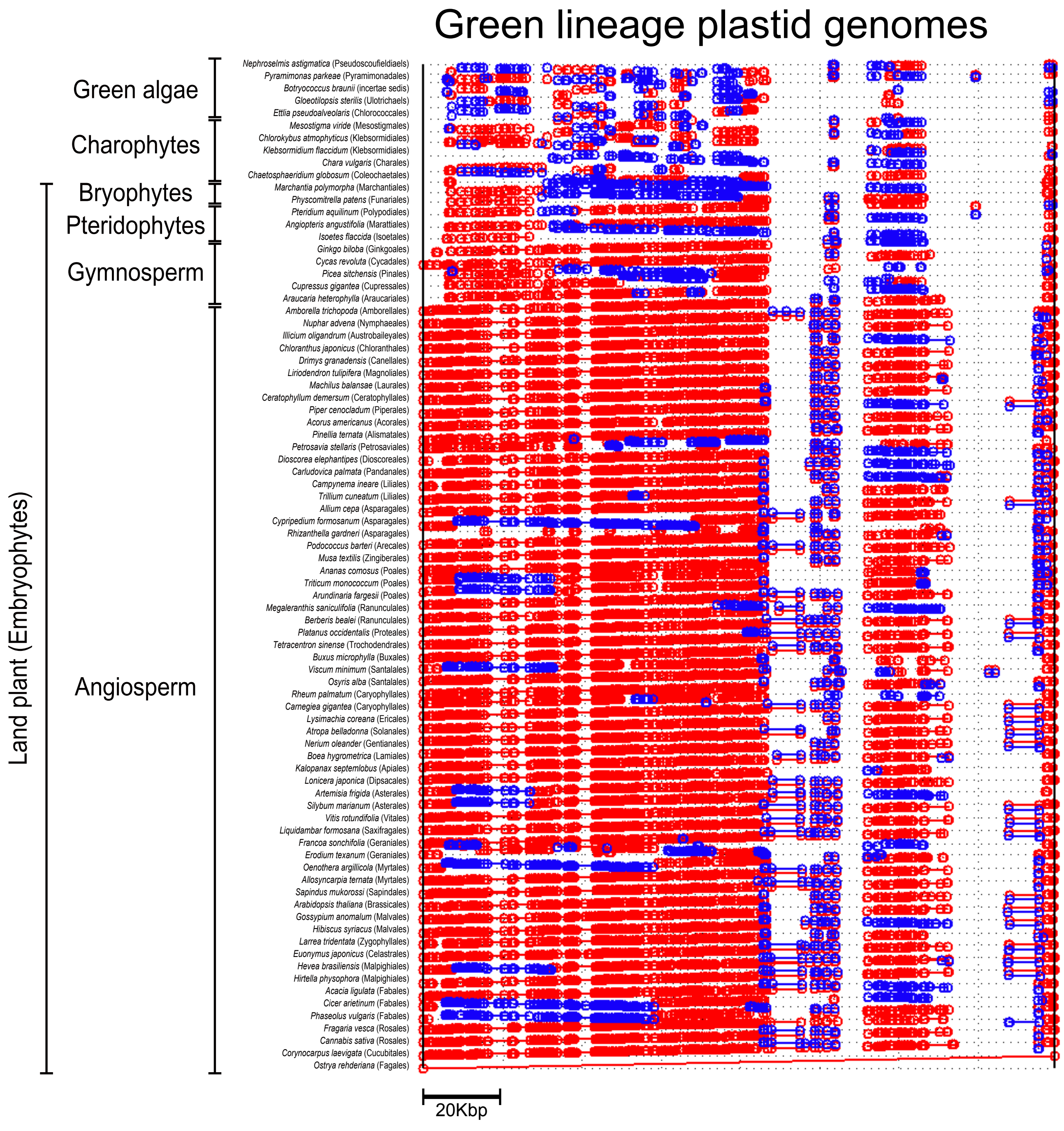

Figure S6. Structural comparison of green lineage plastid genomes based on MUMmerplot result. All plastid genome architectures are compared with the plastid genome of *Ostrya rehderiana* as recent diverged species.

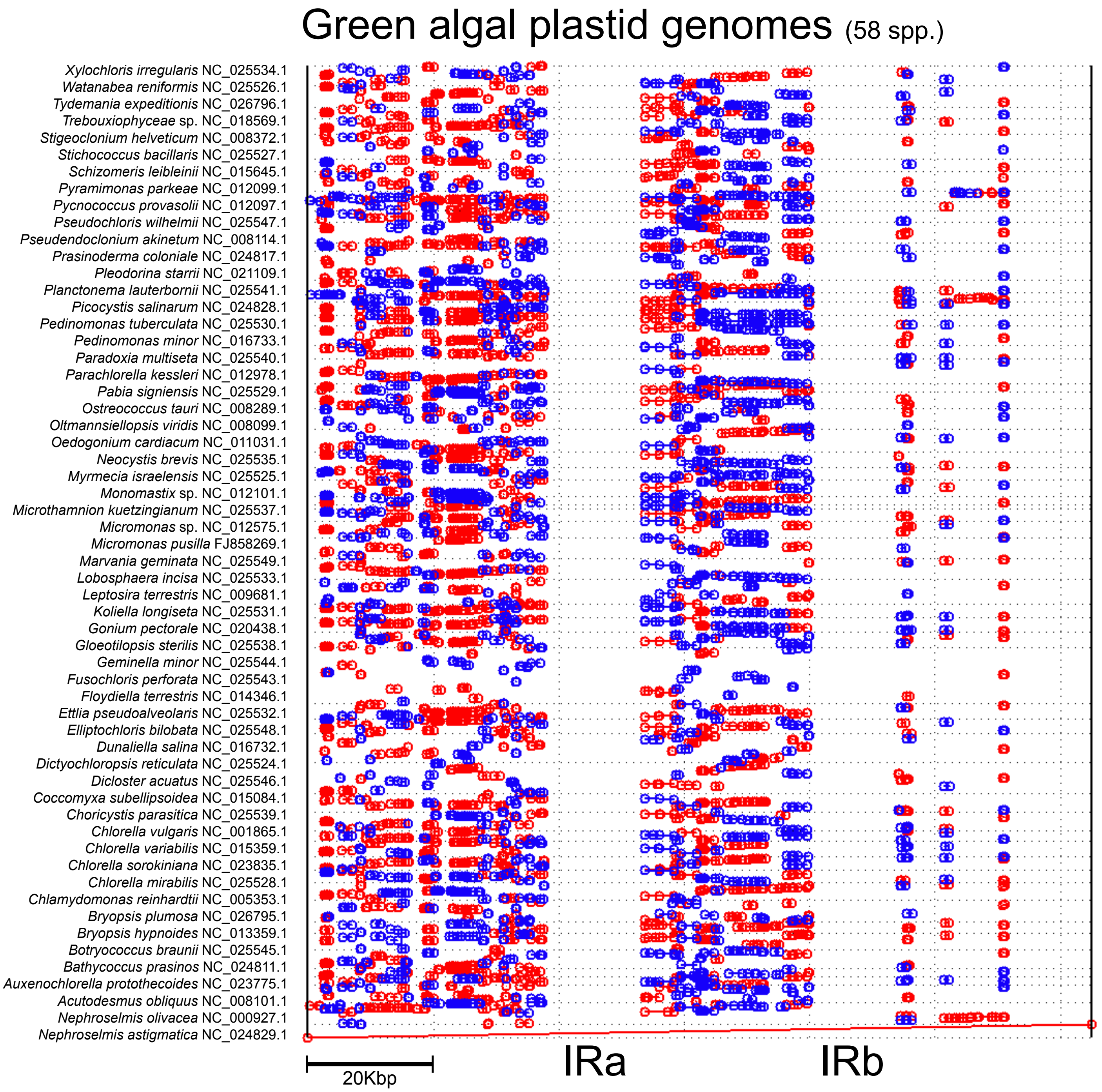

Figure S7. Structural comparison of 58 green algal plastid genomes based on MUMmerplot result. All plastid genome architectures are compared with the plastid genome of *Nephroselmis astigmatica* as one of early diverged species. Empty spaces indicate inverted repeat (IR) regions.

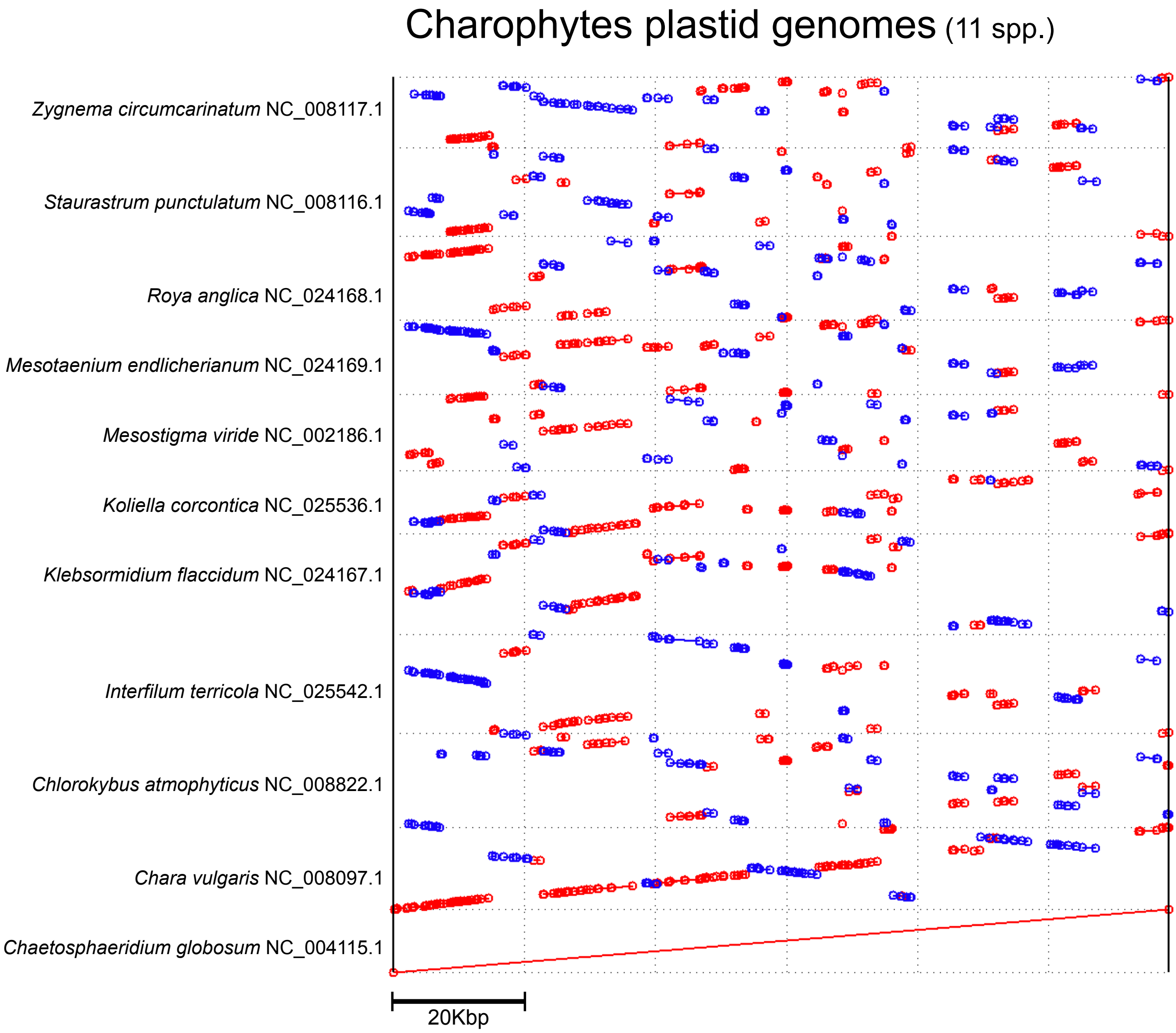

Figure S8. Structural comparison of 11 charophytes plastid genomes based on MUMmerplot result. All plastid genome architectures are compared with the plastid genome of *Chaetosphaeridium globosum* as one of recent diverged species. Empty spaces indicate inverted repeat (IR) regions.

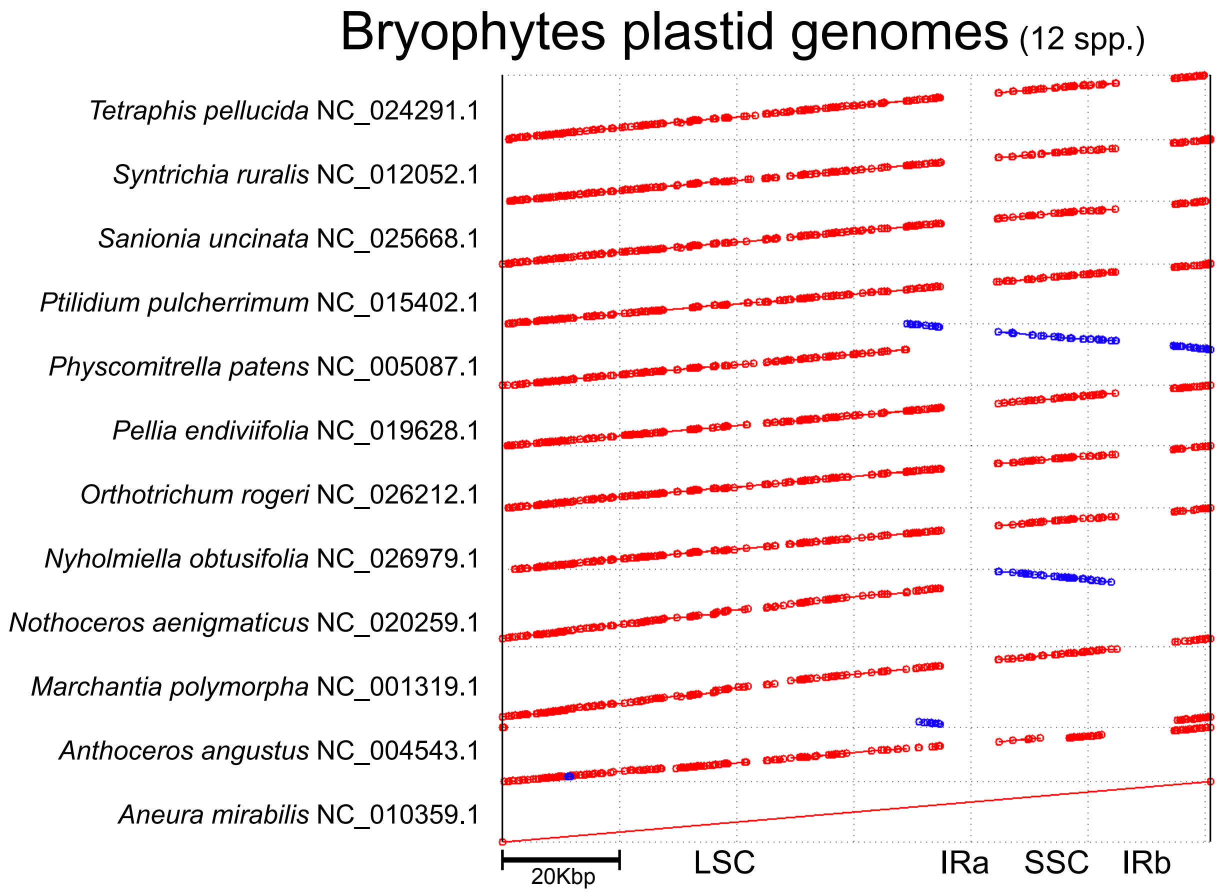

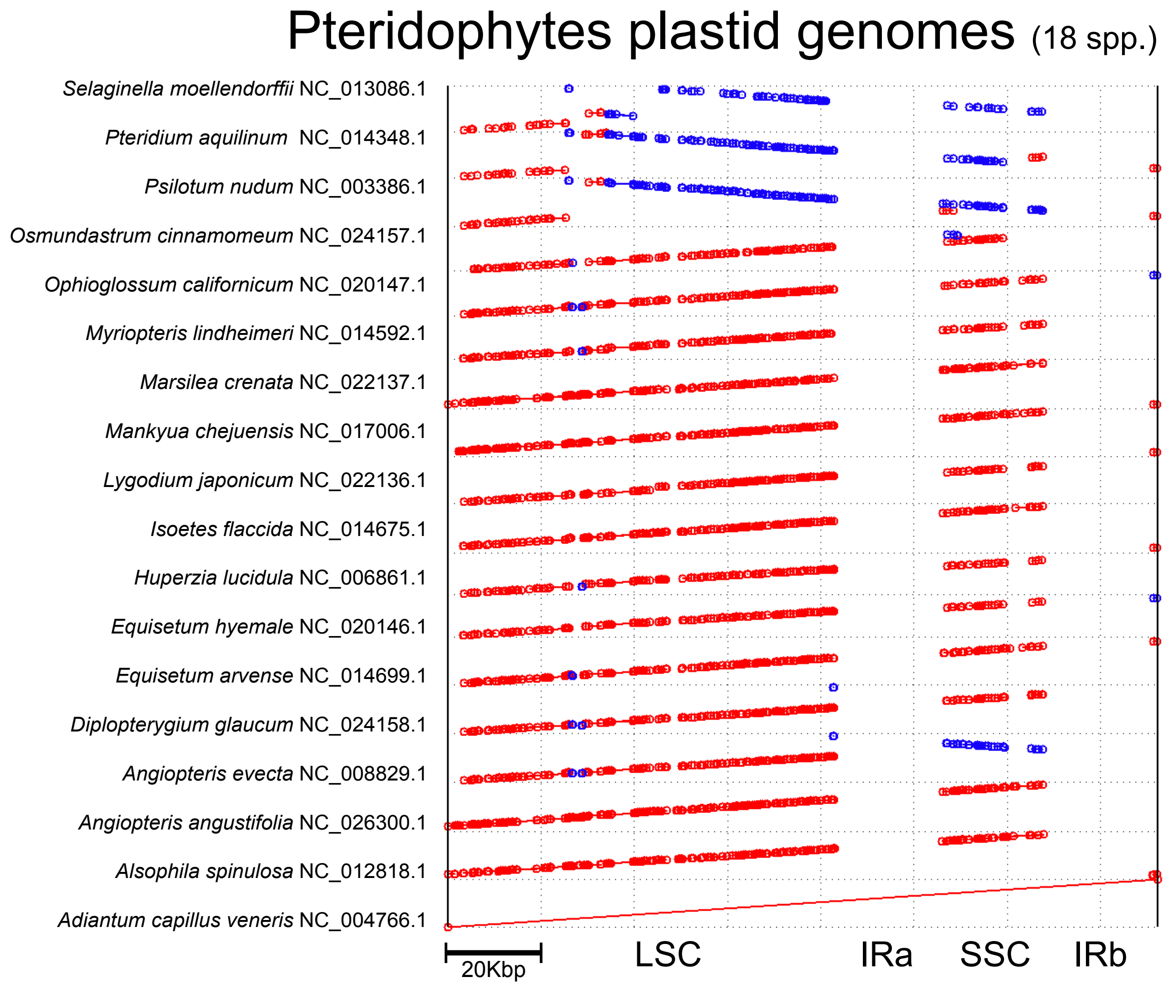

Figure S9. Structural comparison of 12 bryophytes and 18 pteridophytes plastid genomes based on MUMmerplot result. Empty spaces indicate inverted repeat (IR) regions. Between IR regions, there is small single copy (SSC) region in plastid genomes. LSC indicates long single copy region in plastid genomes. All bryophytes and pteridophytes plastid genome architectures are compared with the plastid genome of *Aneura mirabilis* and *Adiantum capillus-veneris* respectively as the most popular genome structure contained species.

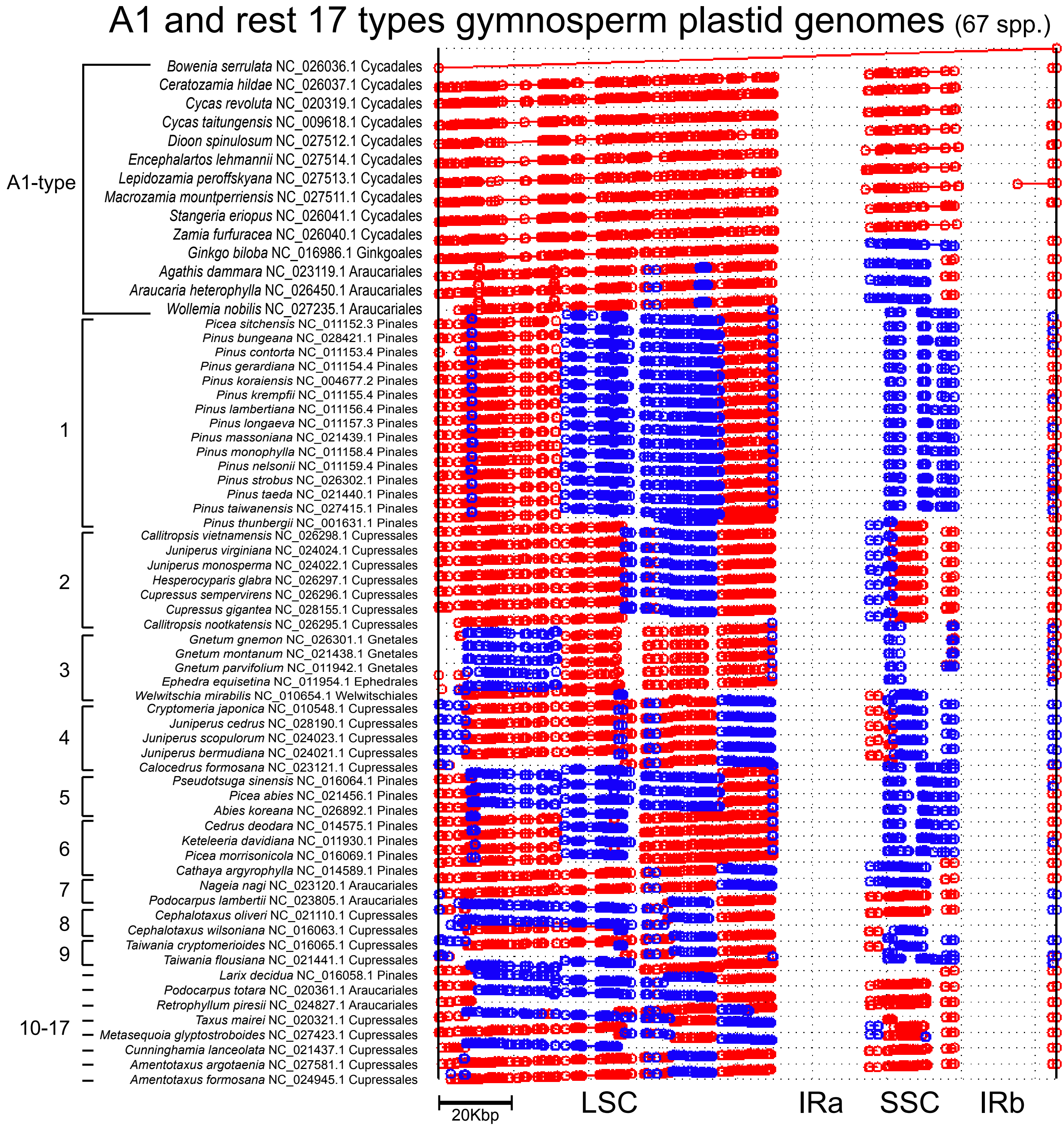

Figure S10. Structural comparison of 67 gymnosperm plastid genomes based on MUMmerplot result. Empty spaces indicate inverted repeat (IR) regions. Between IR regions, there is small single copy (SSC) region in plastid genomes. LSC indicates long single copy region in plastid genomes. All plastid genome architectures are compared with the plastid genome of *Bowenia serrulata* as one of A1-type plastid genome architectures contained species. Fourteen species show A1-type plastid genome architectures.

Type 1 (A1) of angiosperm plastid genomes (437 / 703 angiosperm spp.)

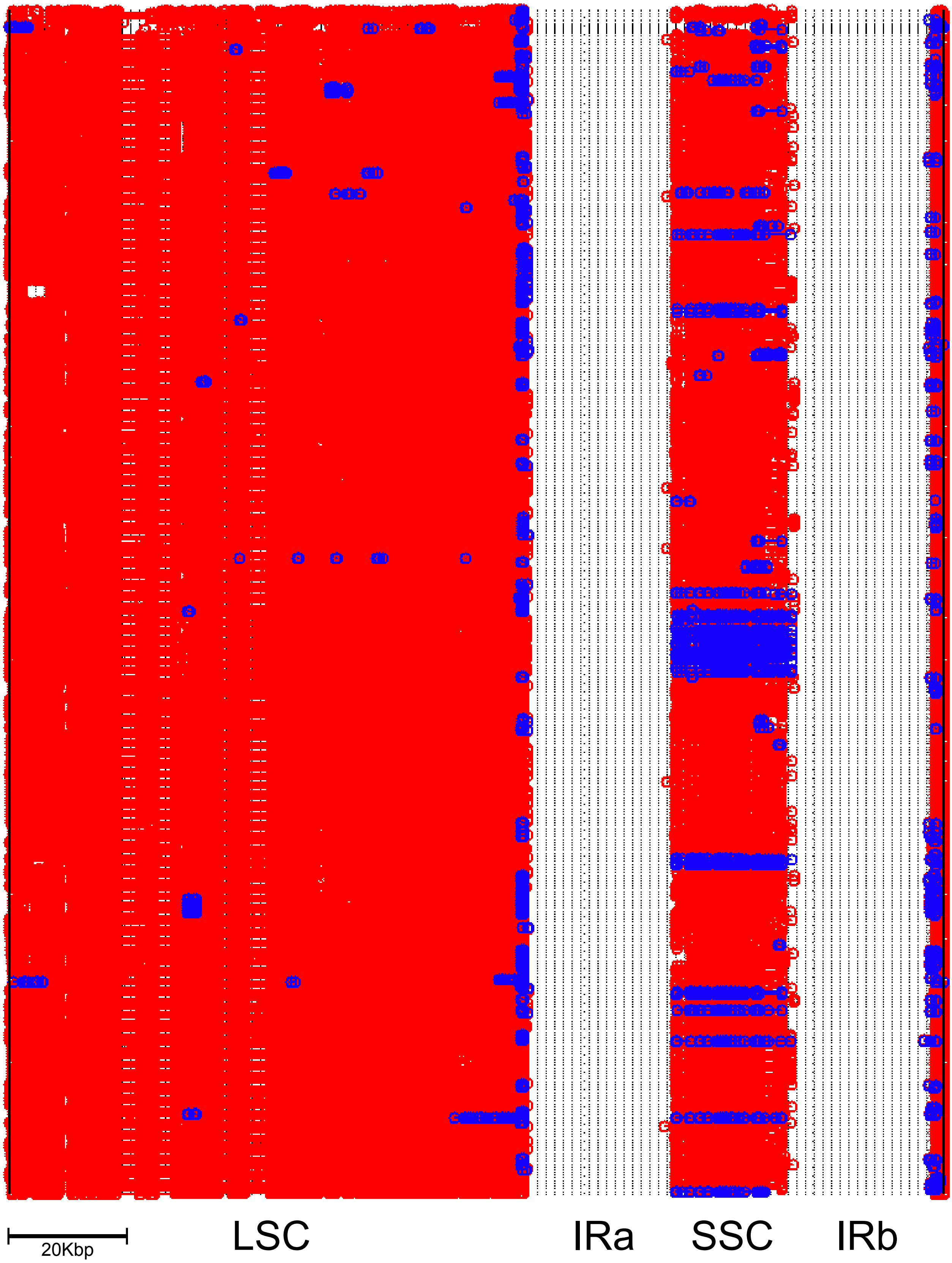

Figure S11. A1-type of angiosperm plastid genomes from MUMmerplot result using 437 angiosperm species. Empty spaces indicate inverted repeat (IR) regions. Between IR regions, there is small single copy (SSC) region in plastid genomes. LSC indicates long single copy region in plastid genomes. All plastid genome architectures are compared with the plastid genome of *Amborella trichopoda* as one of popular genome architecture contained species in angiosperms.

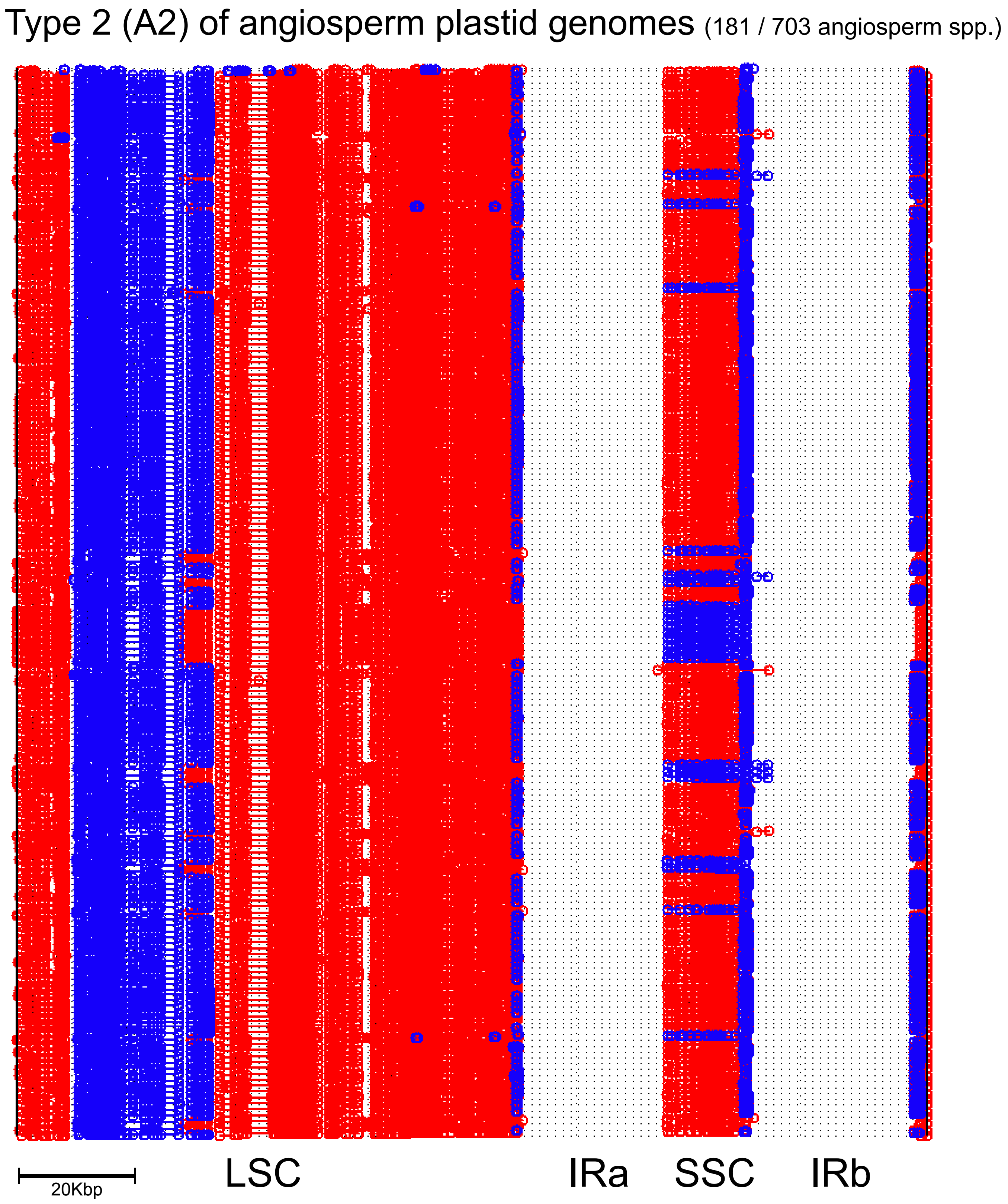

Figure S12. A2-type of angiosperm plastid genomes from MUMmerplot result using 181 angiosperm species. Empty spaces indicate inverted repeat (IR) regions. Between IR regions, there is small single copy (SSC) region in plastid genomes. LSC indicates long single copy region in plastid genomes. All plastid genome architectures are compared with the plastid genome of *Amborella trichopoda* as one of popular genome architecture contained species in angiosperms.

Type 3 (A3) of angiosperm plastid genomes (28 / 703 angiosperm spp.)

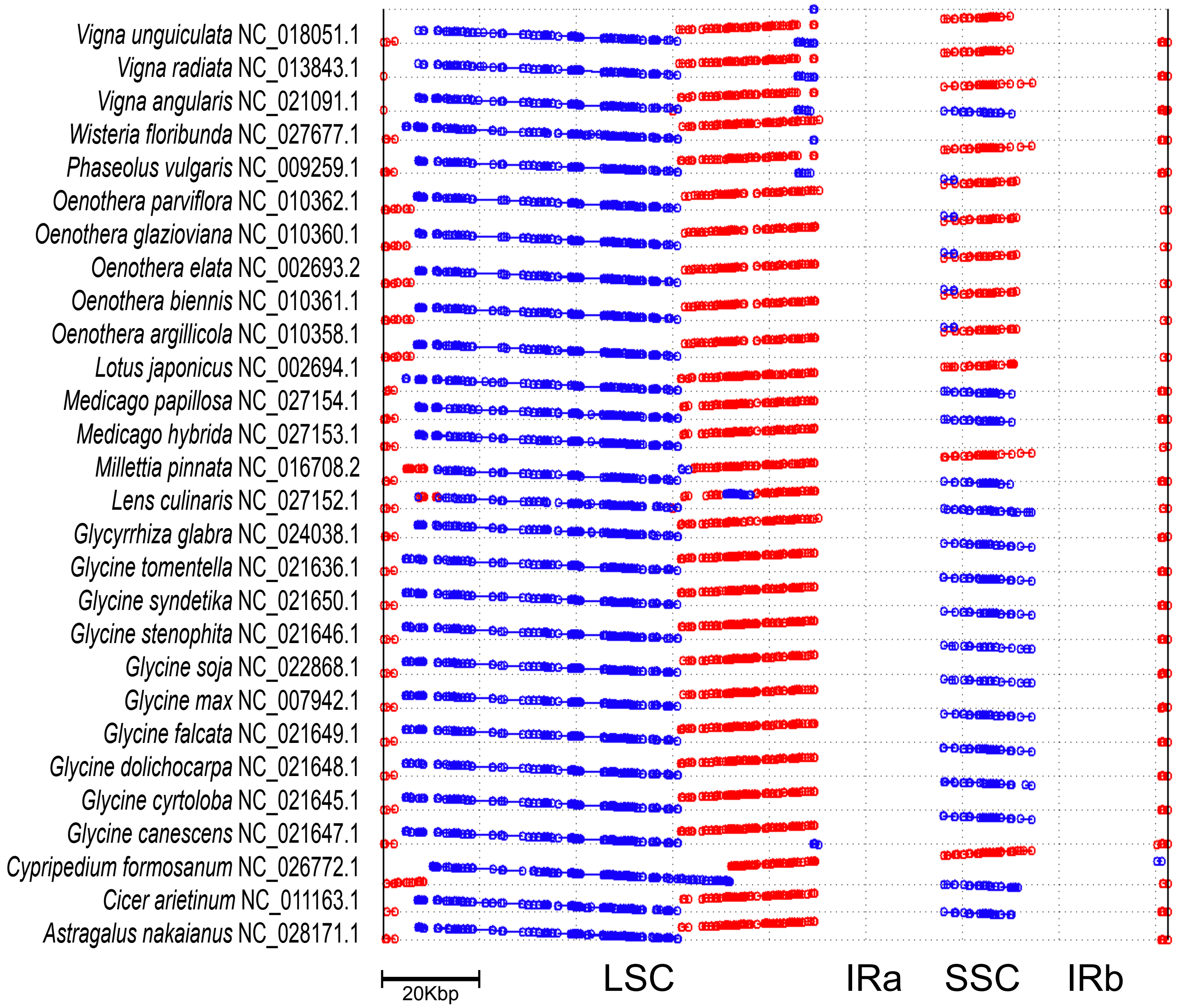

Figure S13. A3-type of angiosperm plastid genomes from MUMmerplot result using 28 angiosperm species. Empty spaces indicate inverted repeat (IR) regions. Between IR regions, there is small single copy (SSC) region in plastid genomes. LSC indicates long single copy region in plastid genomes. All plastid genome architectures are compared with the plastid genome of *Amborella trichopoda* as one of popular genome architecture contained species in angiosperms.

Rest-type of angiosperm plastid genomes (57 / 703 angiosperm spp.)

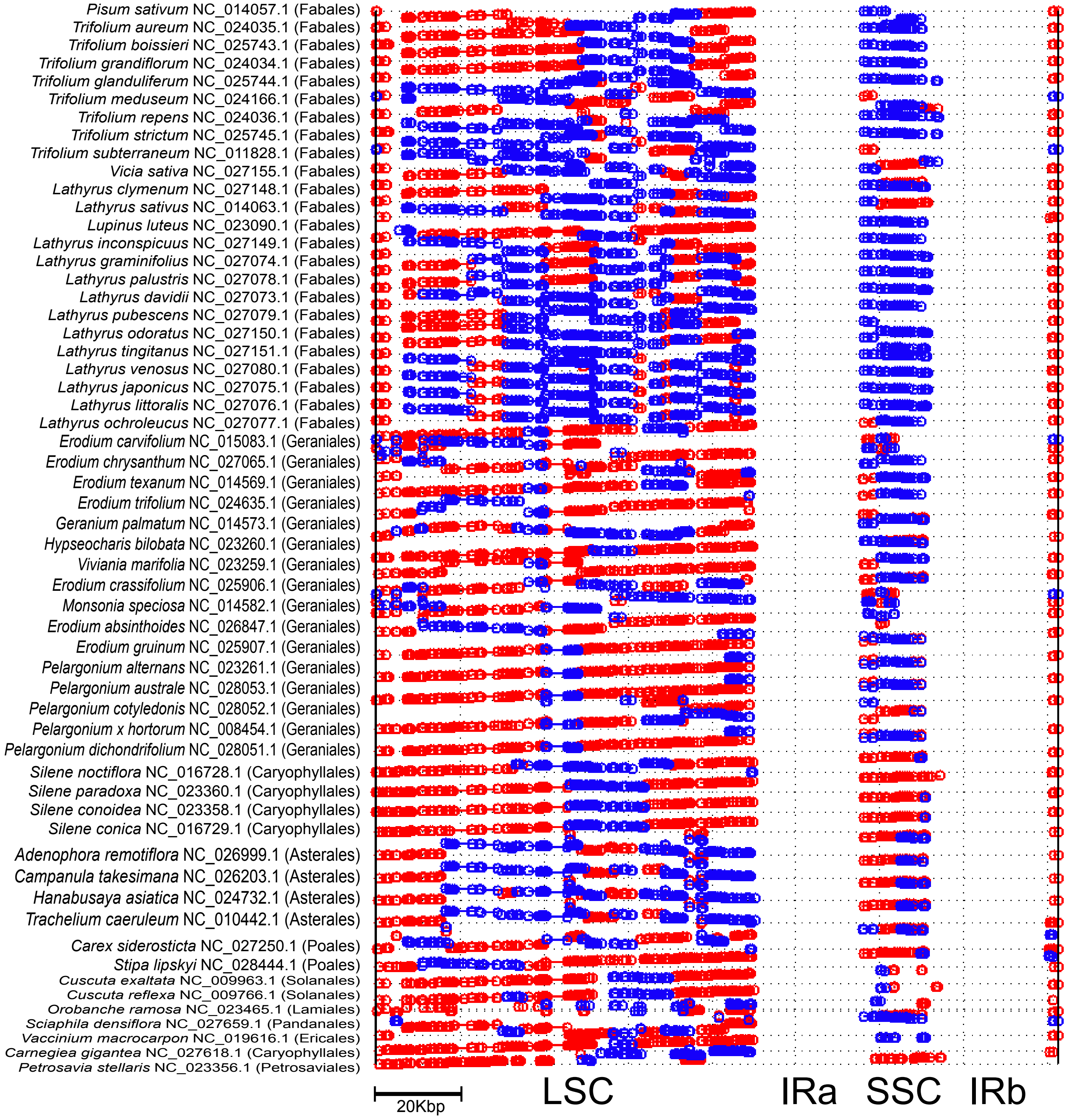

Figure S14. Rest-type of angiosperm plastid genomes from MUMmerplot result using 57 angiosperm species. Empty spaces indicate inverted repeat (IR) regions. Between IR regions, there is small single copy (SSC) region in plastid genomes. LSC indicates long single copy region in plastid genomes. All plastid genome architectures are compared with the plastid genome of *Amborella trichopoda* as one of popular genome architecture contained species in angiosperms.

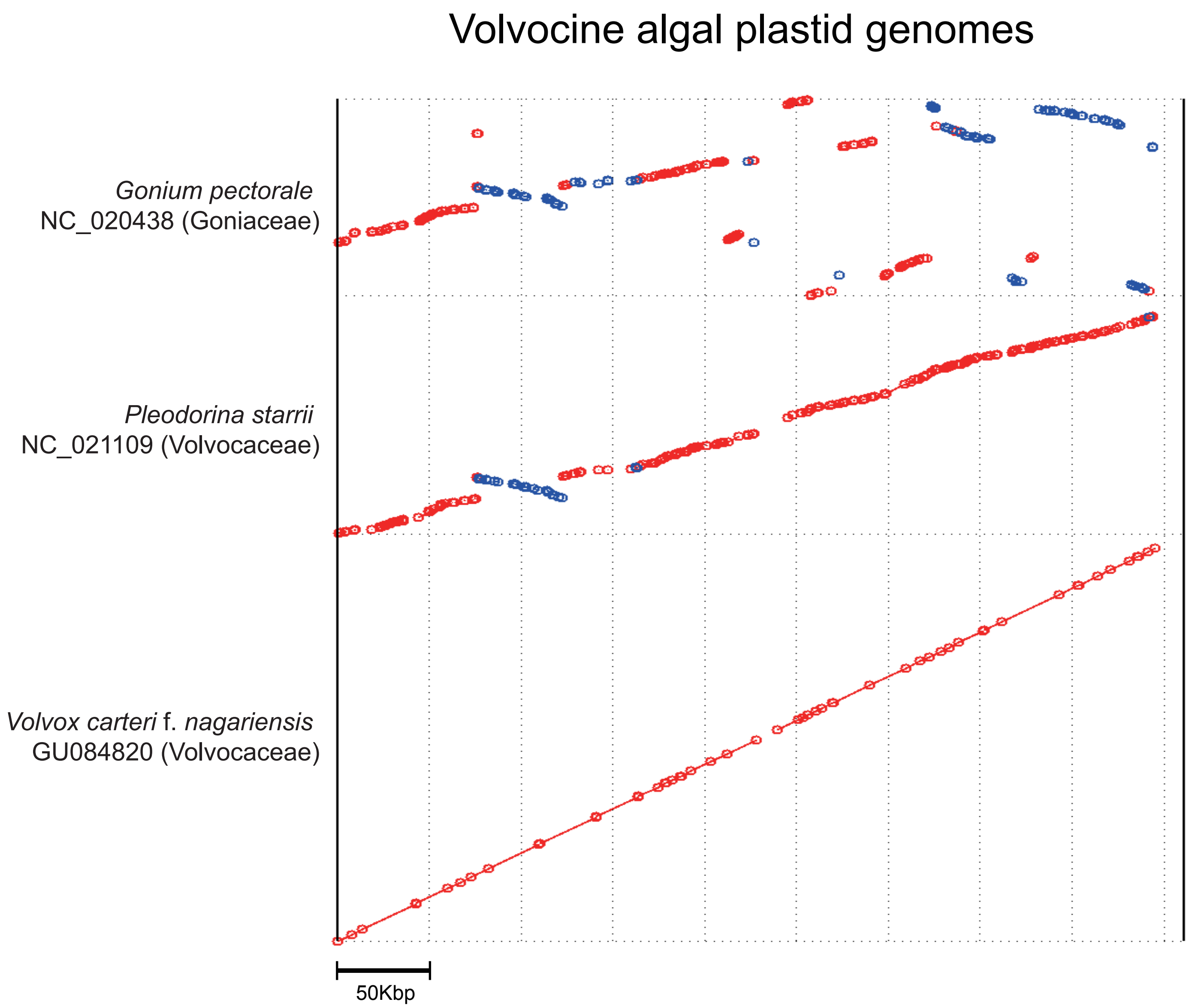

Figure S15. Structural comparison of volvocine algal plastid genomes based on MUMmerplot result. All plastid genome architectures are compared with the plastid genome of *Volvox carteri* f. *nagariensis*.
