# Supplementary material for: Parallel evolution of highly conserved plastid genome architecture in red seaweeds and seed plants
Source: BMC Biol. 2016 Sep 2;14:75. doi: 10.1186/s12915-016-0299-5 (PMC5010701; doi:10.1186/s12915-016-0299-5)
Supplement: Additional file 5: Figure S16. — Structural comparison of Geraniales plastid genomes based on MUMmerplot. (PDF 198 kb) [file 12915_2016_299_MOESM5_ESM.pdf]

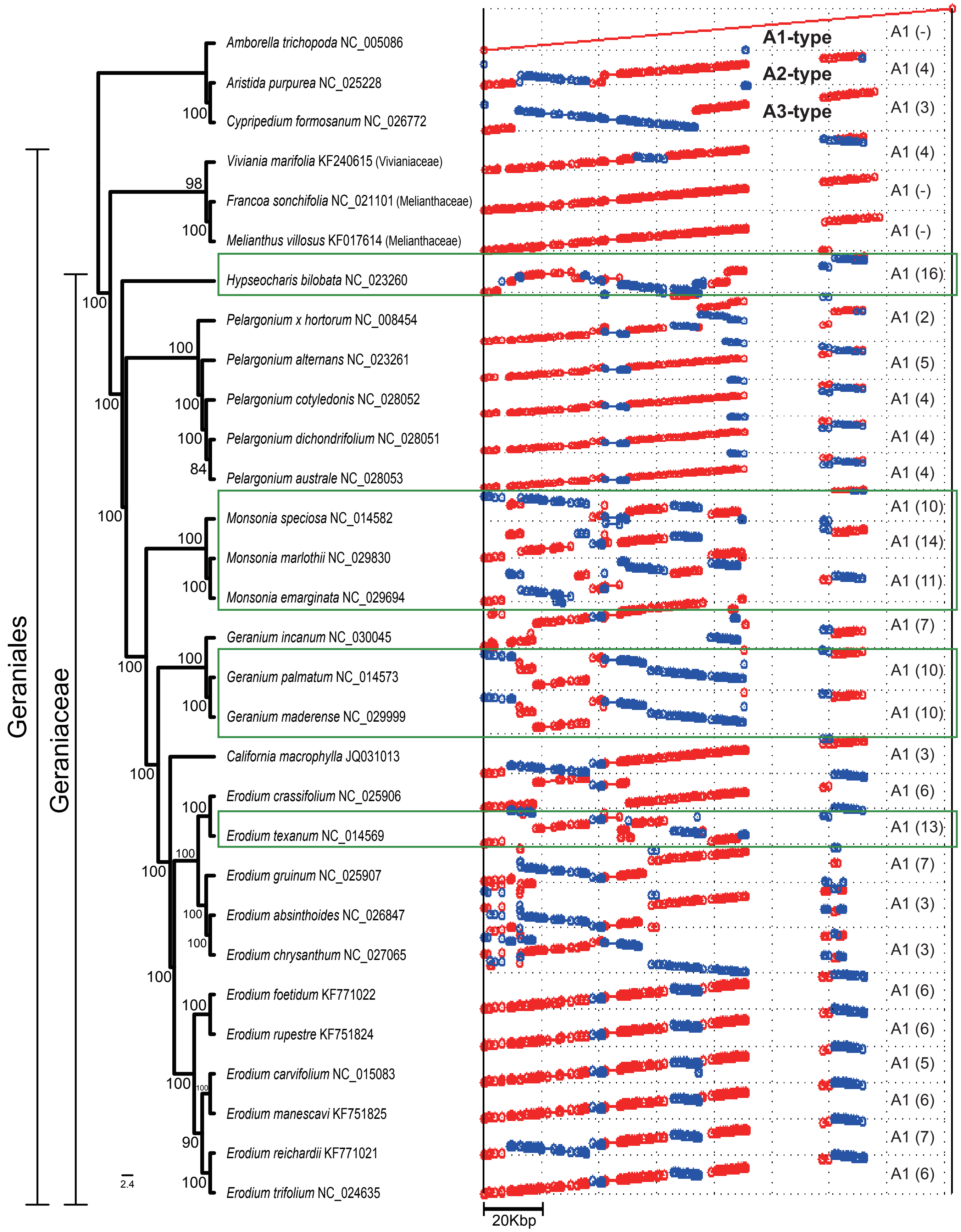

Figure S16. Structural comparison of Geraniales plastid genomes based on MUMmerplot. ML tree built using aligned 75 concatenated proteins from 27 Geraniales plastid genomes (1000 replications, bootstrap support when > 50%) with the outgroup taxa (three popular angiosperm plastid genome architectures). All plastid genome architectures are compared to the A1-type of plastid genome from *Amborella trichopoda.* Breakpoint distances were indicated in parentheses. Low breakpoint distance indicates low variation compared to the A1-type. High architectural variations are marked in green boxes (Breakpoint distance ≥ 10).
