# Supplementary material for: Parallel evolution of highly conserved plastid genome architecture in red seaweeds and seed plants
Source: BMC Biol. 2016 Sep 2;14:75. doi: 10.1186/s12915-016-0299-5 (PMC5010701; doi:10.1186/s12915-016-0299-5)
Supplement: Additional file 6: Figure S17. — Structural comparison of brown lineage plastid genomes based on MUMmerplot. Figure S18. Structural comparison of cryptophyte plastid genomes based on MUMmerplot. Figure S19. Structural comparison of diatom lineage plastid genomes based on MUMmerplot. Figure S20. Structural comparison of Eustigmatophyceae plastid genomes based on MUMmerplot. Figure S21. Structural comparison of haptophytes plastid genomes based on MUMmerplot. (PDF 503 kb) [file 12915_2016_299_MOESM6_ESM.pdf]

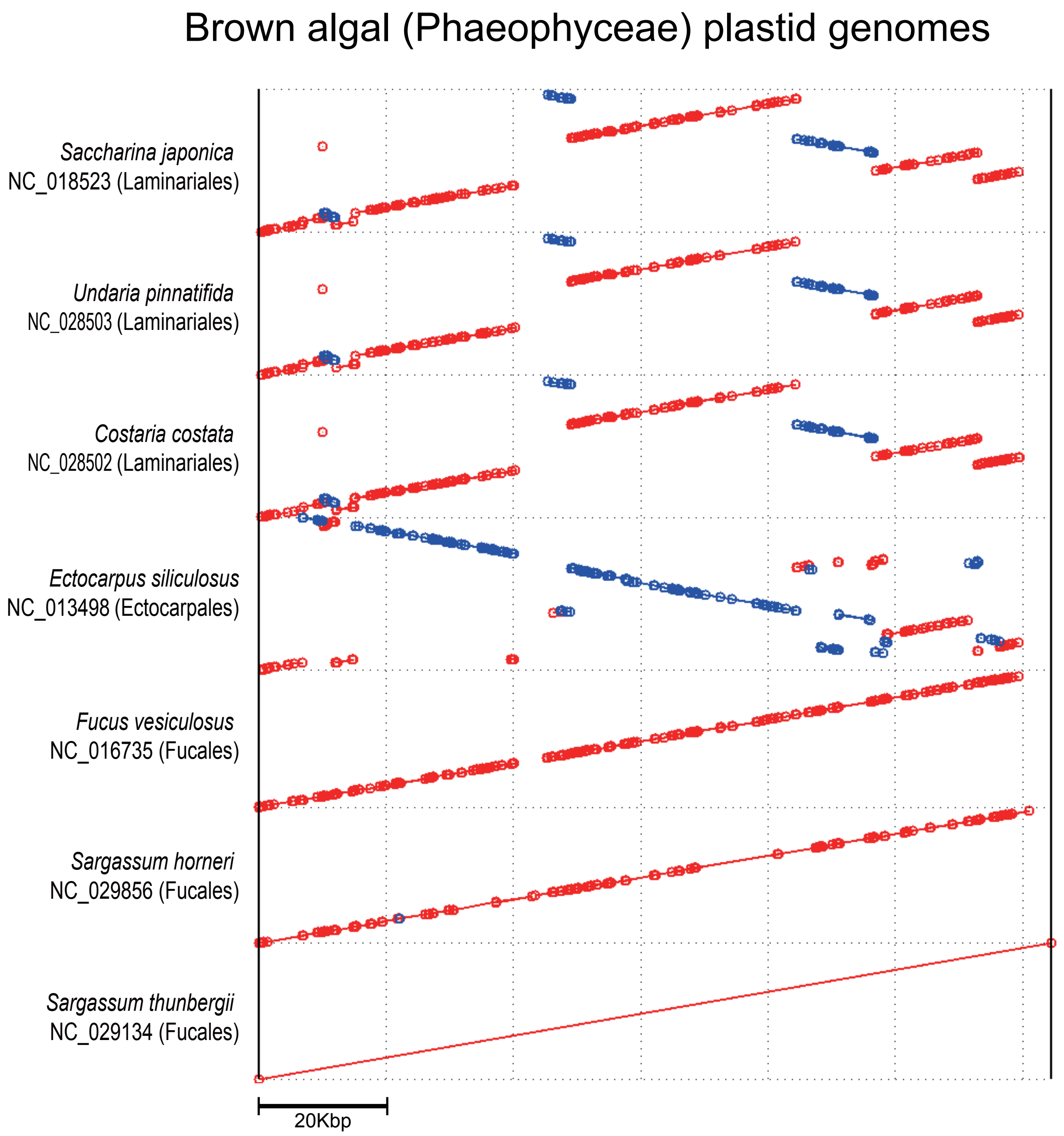

Figure S17. Structural comparison of brown lineage plastid genomes based on MUMmerplot result. All plastid genome architectures are compared with the plastid genome of *Sargassum thunbergii*.

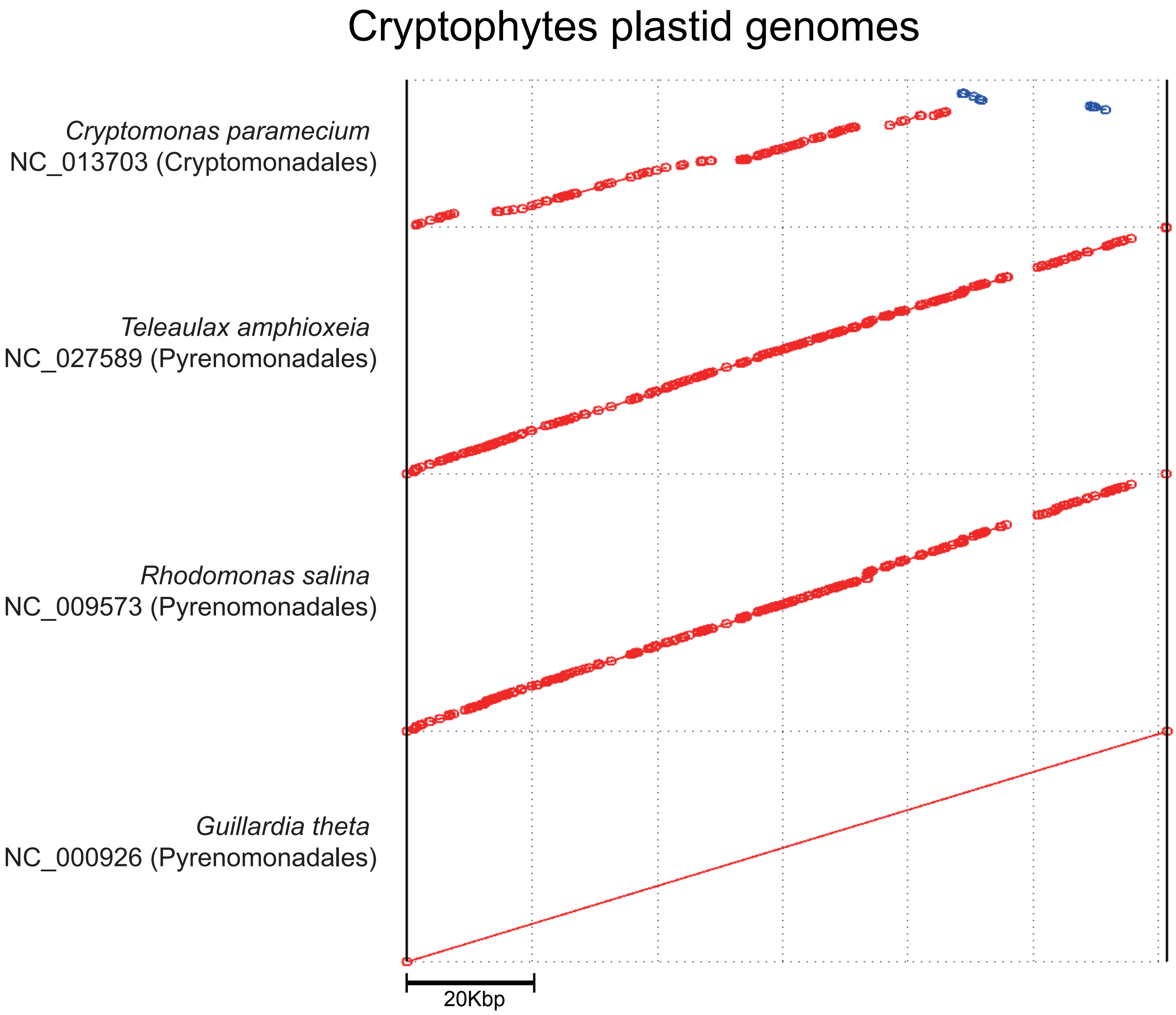

Figure S18. Structural comparison of cryptophytes plastid genomes based on MUMmerplot result. All plastid genome architectures are compared with the plastid genome of *Guillardia theta*.

Diatom (Bacillariophyceae) plastid genomes

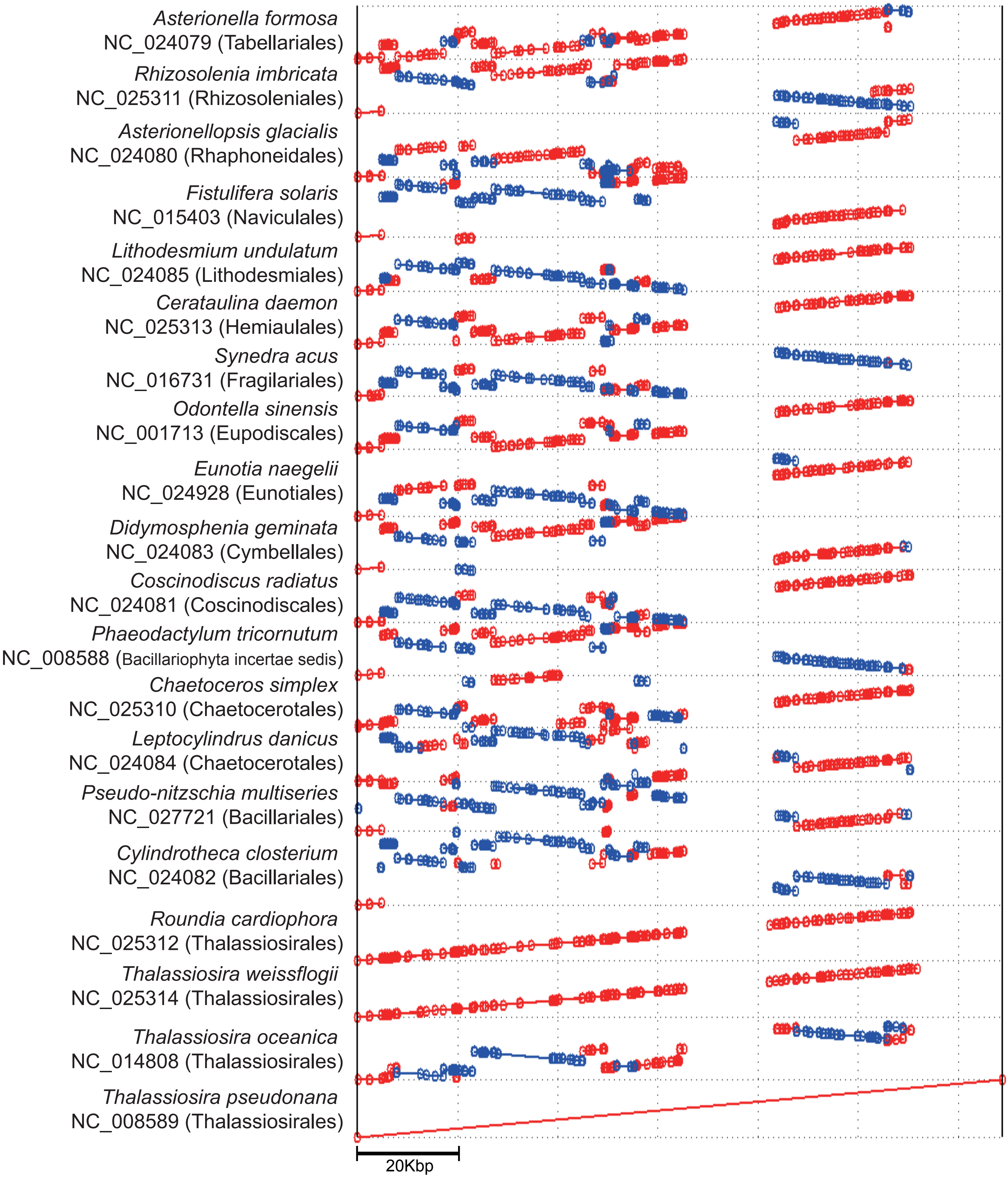

Figure S19. Structural comparison of diatom lineage plastid genomes based on MUMmerplot result. All plastid genome architectures are compared with the plastid genome of *Thalassiosira pseudonana*.

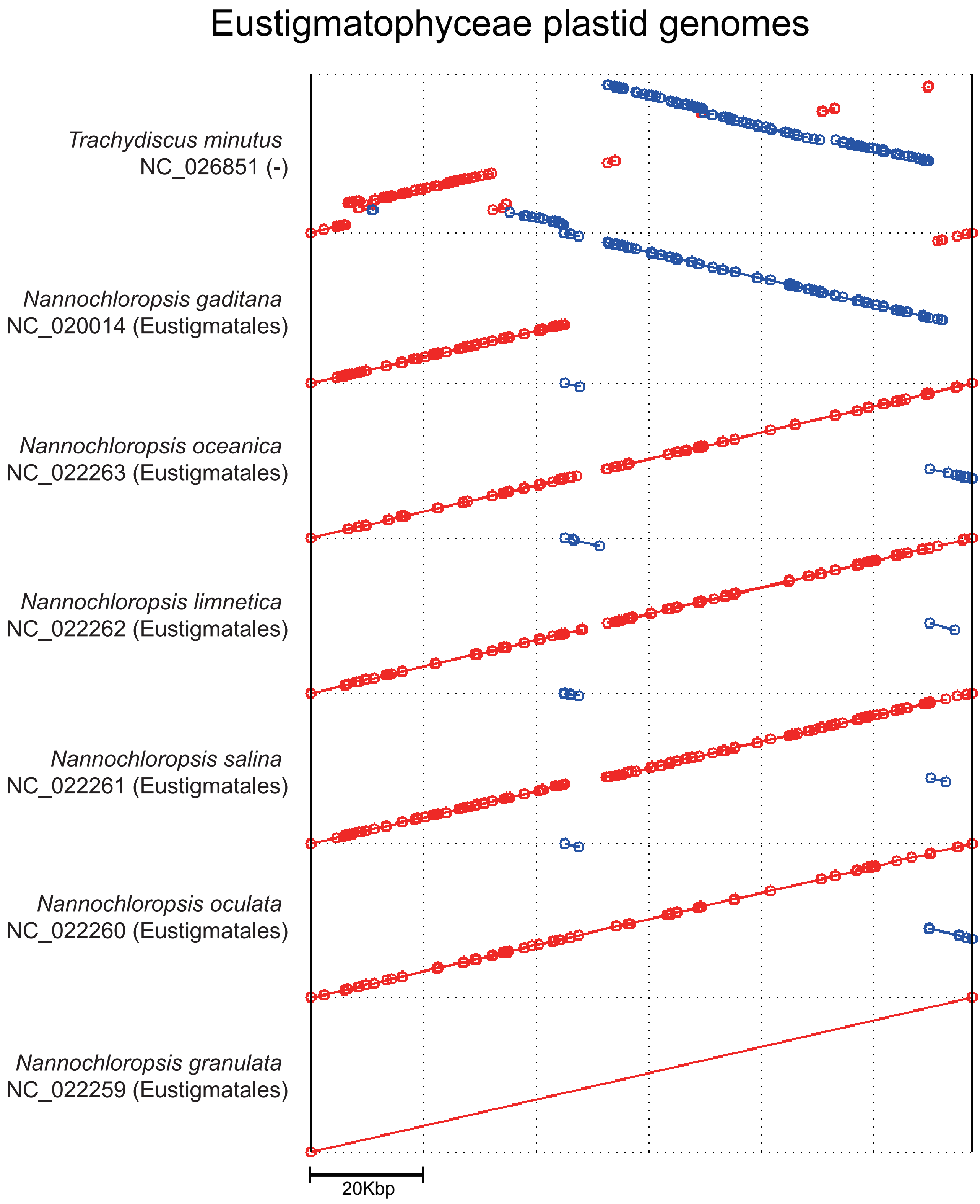

Figure S20. Structural comparison of Eustigmatophyceae plastid genomes based on MUMmerplot result. All plastid genome architectures are compared with the plastid genome of *Nannochloropsis granulata*.

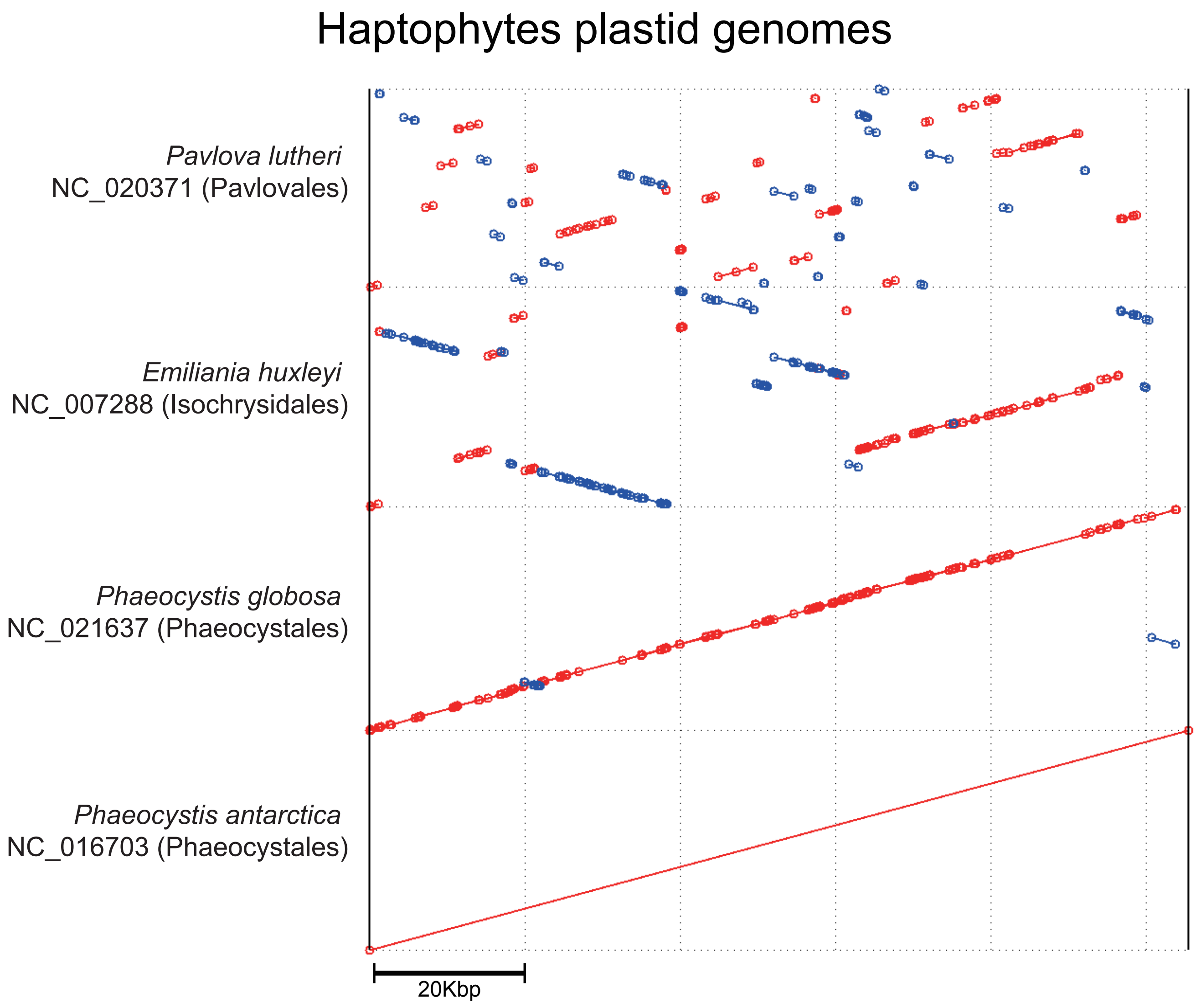

Figure S21. Structural comparison of haptophytes plastid genomes based on MUMmerplot result. All plastid genome architectures are compared with the plastid genome of *Phaeocystis antarctica*.
