# Supplementary material for: Parallel evolution of highly conserved plastid genome architecture in red seaweeds and seed plants
Source: BMC Biol. 2016 Sep 2;14:75. doi: 10.1186/s12915-016-0299-5 (PMC5010701; doi:10.1186/s12915-016-0299-5)
Supplement: Additional file 7: Figure S22. — Box plots (standard deviation) of 770 seed plants and 24 red algal plastid genomes. (PDF 70 kb) [file 12915_2016_299_MOESM7_ESM.pdf]

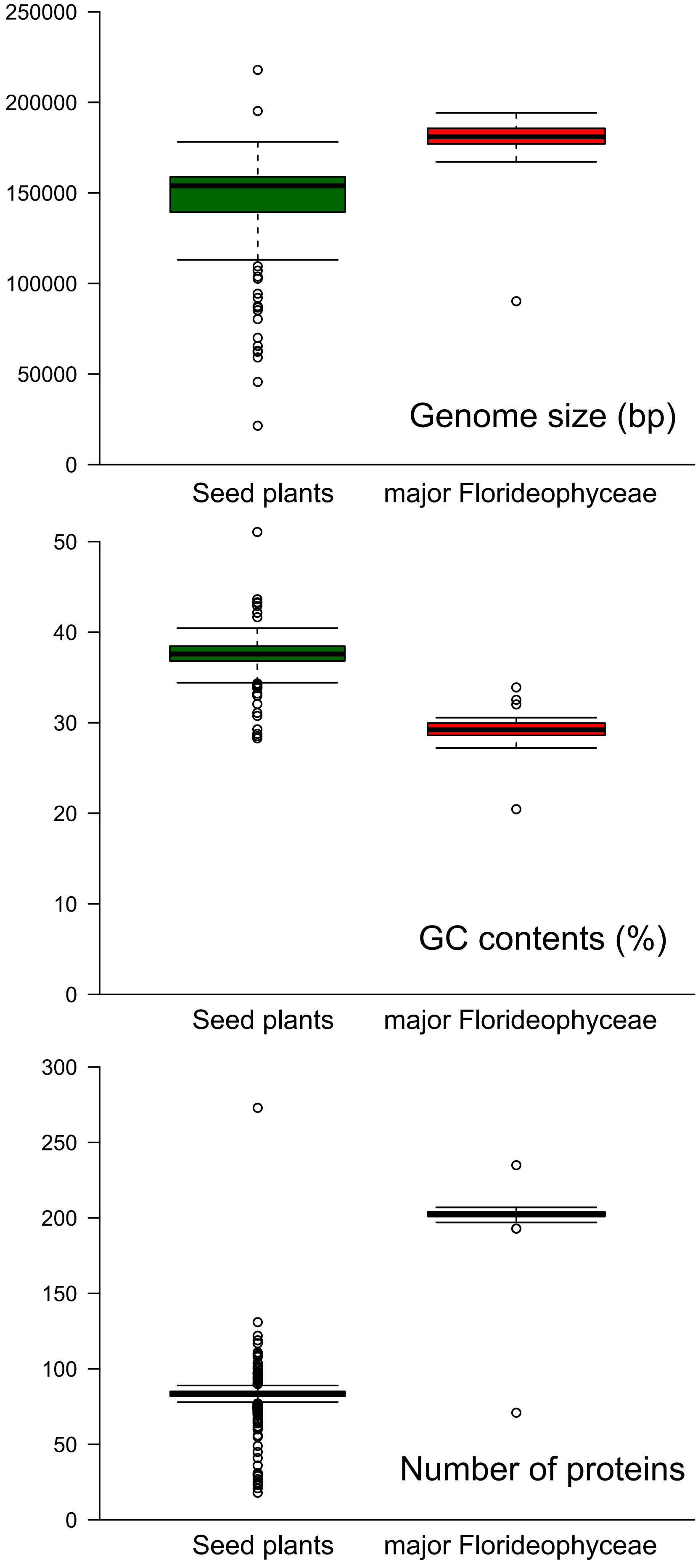

Figure S22. Box plots (standard deviation) of 770 seed plants and 24 red algal plastid genomes. The features are genome size, GC contents and number of proteins from 703 angiosperms, 67 gymnosperms and 24 non-Hildenbrandiophycidae florideophycean species (major Florideophyceae).
