# Supplementary material for: Parallel evolution of highly conserved plastid genome architecture in red seaweeds and seed plants
Source: BMC Biol. 2016 Sep 2;14:75. doi: 10.1186/s12915-016-0299-5 (PMC5010701; doi:10.1186/s12915-016-0299-5)
Supplement: Additional file 8: Figure S23. — ML tree based on concatenated 60 plastid genes from Archaeplastida and their cyanobacterial homologs. (PDF 106 kb) [file 12915_2016_299_MOESM8_ESM.pdf]

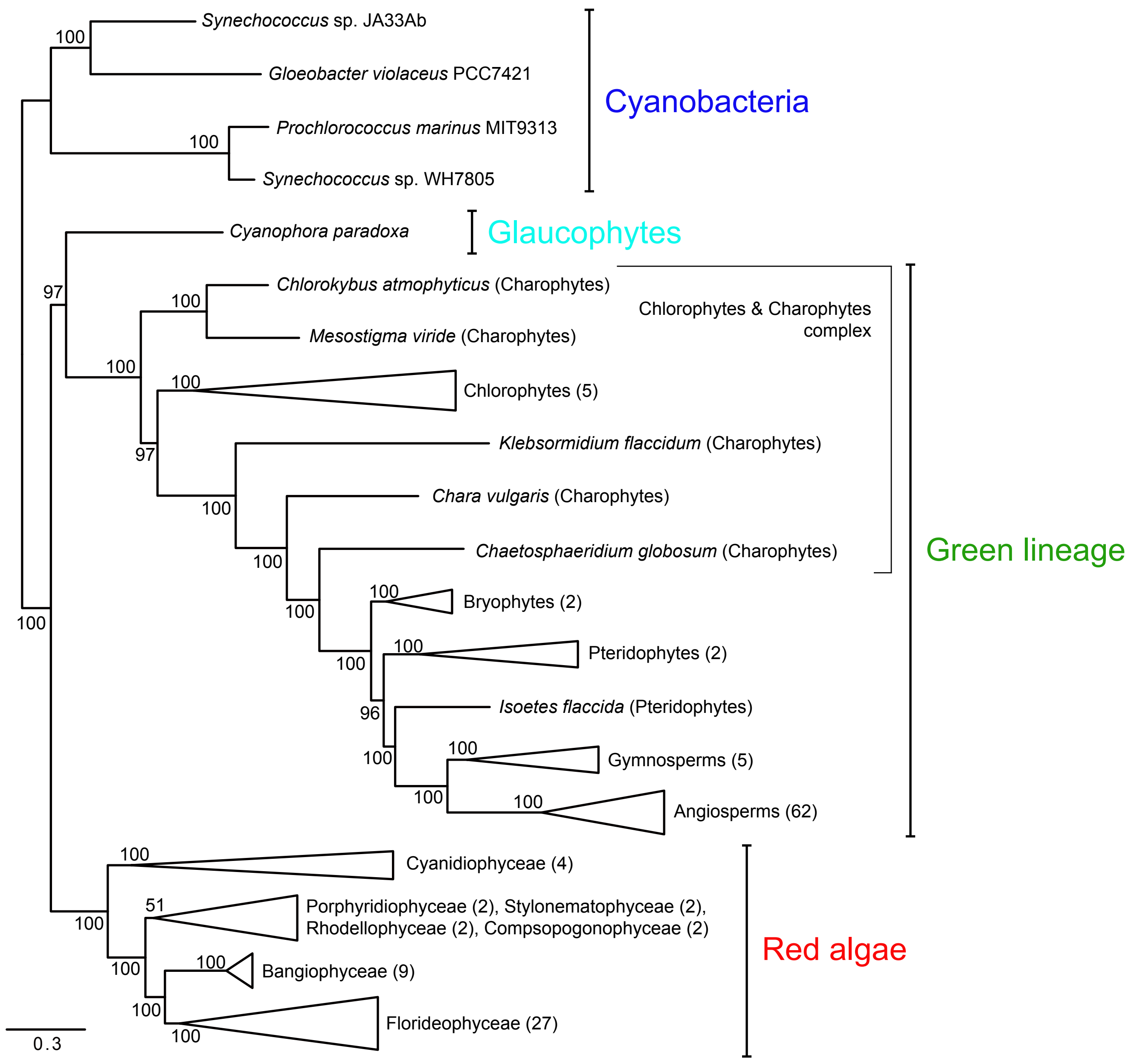

Figure S23. ML tree based on concatenated 60 plastid genes from Archaeplastida and their cyanobacterial homolog genes. 48 red algal species (Fig. 1), 82 green species (Fig. 3), one glaucophyte and 4 cyanobacterial species were used (1000 replications, bootstrap support > 50%).
